# Effective Cross-Link Density as a Metric for Structure–Property Relationships in Complex Polymer Networks: Insights from Acrylic Melamine Systems

**DOI:** 10.1021/acsapm.5c01155

**Published:** 2025-07-17

**Authors:** Amirhossein Gooranorimi, Seyyed Mohammad Mousavifard, Mohsen Mohseni, Hossein Yahyaei, Hesam Makki

**Affiliations:** † Department of Polymer and Color Engineering, 48410 Amirkabir University of Technology, 424 Hafez Avenue, Tehran 15875-4413, Iran; ‡ Department of Chemistry and Materials Innovation Factory, 4591 University of Liverpool, Liverpool L69 7ZD, U.K.

**Keywords:** polymer network, cross-link density, structure−property relationship, molecular simulation, glass transition temperature, network defects

## Abstract

The structural complexities of polymer networks, i.e., multiple functional groups, diverse connection sites, and various defects, make it difficult to accurately describe their microstructure using theoretical models and traditional metrics such as the cross-link density (XLD). This study uses multiscale molecular dynamics simulations to construct complex network structures such as acrylic-melamine systems and establish correlations between their microstructure and thermomechanical properties. By accounting for the elastic contribution of each cross-link point within the network, we modified the XLD and introduced effective XLD (XLD^eff^). Our findings reveal strong linear correlations between XLD^eff^ and both elastic modulus and *T*_g_, relationships that conventional XLD could not establish. This demonstrates the robustness of XLD^eff^ as a predictive metric for thermomechanical properties across diverse cross-linking conversions and prepolymer systems. XLD^eff^ thus serves as a valuable metric for the in silico design and optimization of thermoset polymers with tailored thermomechanical properties.

## Graphical abstract

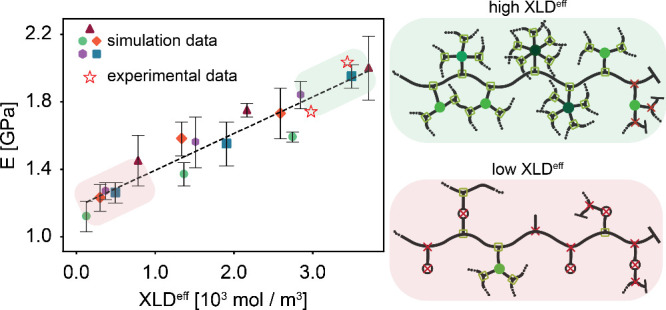

## 1 Introduction

Macroscopic properties of polymer networks are closely linked to their microstructure, which refers to the detailed arrangement of the constituent elements at a microscale,^1,2^ with the network topology being a crucial parameter.^3–8^ Nevertheless, the insolubility of chemically cross-linked polymers makes the experimental characterization challenging, hindering accurate prediction of polymer networks behavior, particularly in systems with multiple connectivity and functional groups. Additionally, several statistical theories have been developed to correlate the network topology and viscoelastic properties. While these models effectively determine average macroscopic properties in many cases, they often assume an ideal network formation and largely overlook the network defects, including various loop orders and free or dangling chains.^9–12^ These defects deteriorate mechanical performance and affect material’s durability by lowering the overall elasticity of the network.

Characterizing the microstructure of polymer networks through experimental techniques is challenging, particularly when it comes to capturing topological features. Among experimental methods, dynamic mechanical analysis (DMA) is a standard technique for quantitative analysis of cross-linked polymers, e.g., measuring cross-link density (XLD). In this method, XLD is estimated by applying classical rubber elasticity theory to the elastic modulus measured in the onset of the rubbery plateau.^13^ However, this approach can be inaccurate for complex networks, as it relies on the assumption of an ideal defect-free network structure, which becomes unreliable when the network contains many defects.^14,15^ The distribution and presence of defects in the network significantly change the thermomechanical properties, especially in complex systems.^4,15–18^ More advanced techniques, such as network disassembly spectrometry and multiple-quantum NMR, are available for quantifying defects;^19–21^ however, like many other experimental and theoretical methods, they primarily focus on systems involving end-link prepolymers and cannot be generalized to networks made from prepolymers and cross-linkers with diverse functionalities and connecting sites along the chains. Therefore, analyzing complex networks solely through experimentation and theoretical approaches is challenging, and molecular modeling can be an effective complementary tool.^22–27^

Molecular dynamics (MD) simulation is among the most successful methods for modeling polymer networks through a reactive scheme.^28,29^ It accounts for the stiffness of prepolymer chains through explicit force field parameters such as angular and torsional potentials, steric hindrance of surrounding chains, which affect reactive groups accessibility, and the evolution of the spatial arrangement of chains and cross-linkers during the reaction. Therefore, various researchers employed MD-based reactive schemes to construct a reliable network structure, able to predict many properties that were in good agreement with the experimental results.^13,14,30–35^

While atomistic simulations provide full molecular detail, they are often limited in accessible system sizes and time scales, making long-range equilibration and reaching high reaction conversions computationally challenging.^36,37^ In contrast, coarse-grained (CG) modeling enables (i) long-range equilibration, (ii) reaching higher reaction conversions, comparable to experimental levels, and (iii) significantly longer simulation times.^38–41^ Nonetheless, they come short in representation of the (local) segmental dynamics and accurate calculation of thermo-mechanical properties, which heavily depend on atomistic interactions.^42,43^ Therefore, multiscale modeling is a viable solution for combining the benefits of both CG and atomistic levels, so that the cross-linking reactions can be performed at the CG level and further equilibration and property calculations can be done after reverse-mapping the CG cross-linked models to the atomistic representation.^44–47^

Using a recently developed polymerization package (PolySMart),^38^ and adopting a reverse-mapping methodology,^48^ this paper aims to introduce a more comprehensive network characteristic for complex cross-linked polymers formed from multifunctional prepolymer chains and cross-linkers, such as those found in acrylic-melamine. We demonstrate that standard parameters like reaction conversion and XLD lack the accuracy and overlook network features needed to predict thermo-mechanical properties. Accordingly, we introduce a single network parameter by modifying XLD, namely XLD^eff^, which directly links the microstructure and thermo-mechanical properties. Furthermore, the results of our multiscale simulations- validated against experimental data- explore the role of functionality and prepolymer chain length at various stages of cross-linking.

## 2 Method

### 2.1 Simulation

#### 2.1.1 Molecular Structures and System Compositions

We use five distinct acrylic prepolymers divided into two main categories: (i) structures with the same chain length but variable functionality per unit mass, i.e., all medium chain length, M-F1 (low OH functionality: 83 mg KOH/g resin), M-F2 (medium OH functionality: 103 mg KOH/g resin), and M-F3 (high OH functionality:142 mg KOH/g resin), and (ii) structures with different chain lengths but the same functionalities per unit mass, i.e., S–F3 (short chain with *M*_w_ ≈ 1382 g/mol), M-F3 (medium chain with *M*_w_ ≈ 2765 g/mol), and L-F3 (long chain with *M*_w_ ≈ 5552 g/mol). In the S–F3 model, two chains (with the same molar ratio) are used to maintain a constant hydroxyl/carboxyl ratio for all models. All precursors are cross-linked with hexa­(methoxymethyl)­melamine (HMMM) in a 1:1.2 molar ratio of reactive groups, with excess melamine used to align with common industry practice (see the structures in Figure 1a and reactions in Figure 1b). Table 1 summarizes the details of five different systems. A snapshot from the equilibrated model (Figure 1c) and the melamine-melamine radial distribution function (RDF_mel‑mel_), excluding intramolecular interactions, show small local melamine aggregates (Figure 1d), which is an expected occurrence in acrylic-melamine mixtures.^49^ This is an expected occurrence in acrylic-melamine mixtures because melamine is slightly more polar and has an intrinsic tendency to self-condense^50–52^ The structure and properties of the polymers are investigated during the curing process (detailed in SI, Section S3, along with Figures S18–S24). Note that in this study, we adopt a multiscale strategy, using CG simulations to construct cross-linked networks, followed by reverse mapping to atomistic structures for further equilibration and accurate thermo-mechanical analysis.

**Table 1:** Five Model Structural Characteristics

system	number of acrylic-to-melamine molecules	*M*_w_ of acrylic [g/mol]	number of prepolymer’s beads	theoretical OH value (functionality per unit mass) [mg KOH/g]
M-F1^a^	640–512	2714 ≈ 2X	74	83
M-F2	626–626	2730 ≈ 2X	74	103
M-F3	600–840	2765 ≈ 2X	74	142
S-F3	1200–840	1382 = X	36 and 38	142
L-F3	300–840	5552 ≈ 4X	148	142

a There are three distinct functionality types (F1, F2, F3) corresponding to low, medium, and high OH functionality, and three different chain lengths (S, M, L) corresponding to short, medium, and long chain lengths.

**Figure 1:**
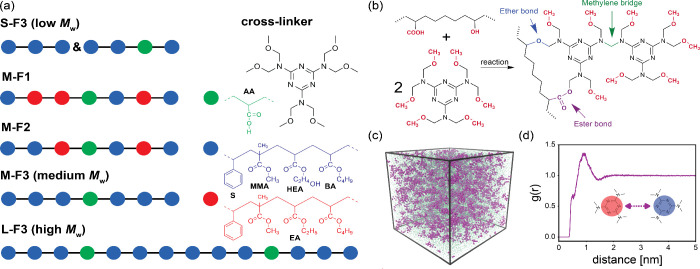
(a) Structure of acrylic prepolymers and cross-linker (HMMM). Monomers used in acrylic resin include styrene (S), methyl methacrylate (MA), 2-hydroxyethyl acrylate (HEA), butyl acrylate (BA), and acrylic acid (AA). Ethyl acrylate (EA) monomer has been used instead of HEA monomer in the structures where the hydroxyl value is changed. The colors are used to emphasize compositional differences and do not represent the coarse-grained model. For bead typing and detailed chemical structures, see Figures S5 and S6 in the SI. (b) Schematic of cross-linking reaction with reactive groups shown in red, newly formed ester bonds in purple, ether bonds in blue, and methylene bridges from melamine self-condensation in green. (c) Snapshot of the simulation box (melamine and acrylics are shown in purple and green, respectively). (d) The radial distribution function of melamine-melamine before initiating the cross-linking reaction (intramolecular interactions were excluded).

#### 2.1.2 Force Field Parametrizations

To develop an accurate CG model and enable reverse mapping, atomistic simulations were first performed on the individual components of the network. Atomistic models of acrylic prepolymer and hexa­(methoxymethyl)­melamine (HMMM) were first optimized using DFT calculations (B3LYP/6–31G*) to compute partial charges via CHELPG method, while other force field parameters were obtained from OPLS-AA.^53^ The bulk properties of these molecules, i.e., density, chain end-to-end distance, and radius of gyration, were calculated from the equilibrated melt models using GROMACS 2022 ^54^ under NPT conditions at 300 K and 1 bar to serve as references for CG parametrization and reverse mapping. Full details are provided in Section S2.1 of the SI. Then, CG models were constructed following the Martini 3 ^55^ protocol by mapping atomistic structures onto CG beads. Bonded parameters derived from atomistic trajectories and nonbonded parameters were directly taken from MARTINI 3 force field, according to the bead typing provided in SI, Section S2.2.1. The CG model was validated against atomistic simulations by comparing essential parameters, such as the radius of gyration, end-to-end distance, and density, which showed quantitative agreement with a maximum deviation of 5%. These parameters are important indicators of bonded and nonbonded interactions that reflect the material’s overall behavior. Full methodological details, including force field parameters and validation results, are provided in the SI (Sections S2.2, S.2.3, and S.2.5, along with Equations SE1–S5 and Tables S3–S6).

#### 2.1.3 Cross-Linking Simulation

In CG level, before initiating the cross-linking reactions, acrylic and melamine molecules were mixed, and systems were equilibrated for 150 ns at 300 K and 1 bar. Cross-linking reactions were simulated using the PolySMart tool,^38^ which incorporates reaction probability and distance-based criteria to form bonds between reactive beads. PolySMart enables step-growth polymerization by iteratively identifying reactive pairs within a predefined cutoff distance, forming new bonds based on user-defined probabilities, and updating system topology, i.e., force field files, accordingly, followed by MD relaxation steps (a short 50 ps equilibration with 1 fs time step, and then 1 ns equilibration with 5 fs time step to fully relax the newly formed bonds). This iterative process continued until the target conversion level of ≈ 85% was achieved. At each stage, structure and topology files were saved, allowing for detailed analyses of network evolution at the molecular level. Further details are provided in the SI (Sections S2.4 and S2.5, along with Figures S7–S9 and Tables S7–S10).

#### 2.1.4 Reverse Mapping to Atomistic Scale

The cross-linked structures obtained from CG simulations were reverse-mapped to atomistic scale using the Martini back-mapping framework^48^ to accurately evaluate thermo-mechanical property. The CG topologies were first converted to atomistic OPLS-AA format, followed by structure generation using the standard Martini reverse-mapping protocol. A multistep relaxation process was performed under NVT conditions with gradually increasing time steps (0.1 to 1 fs), ensuring stable and relaxed atomistic structures for subsequent property evaluations. Details of the reverse-mapping procedure are provided in the SI, Section S2.6, along with Figures S10 and S11.

#### 2.1.5 Thermomechanical Properties Calculations

To simulate the tensile test in MD, we use a standard procedure^57^ on the equilibrated atomistic structures. Simulation boxes are stretched in the Z direction, keeping the pressure constant (1 bar) in the X and Y directions at the temperature of 300 K. Elastic modulus is obtained from fitting the data in the range of 2–5% of strain of the stress–strain curve (see Figure 2a). Details of tensile simulation are provided in the SI, Section S2.7.1, along with Figures S12.

**Figure 2:**
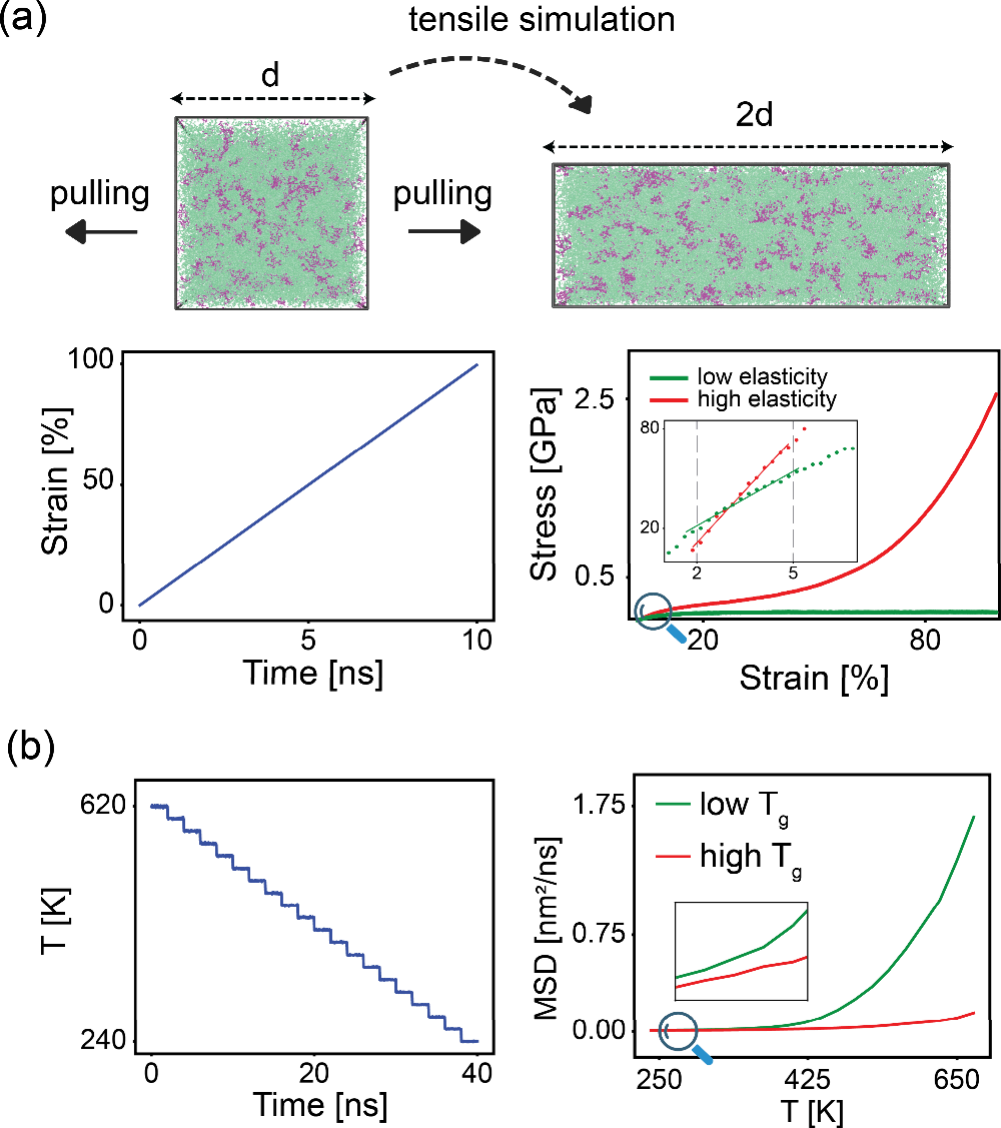
(a) Schematic representation of tensile simulation. The box was pulled in the Z direction to 100% strain (deform rate *≈* 1.36 nm/ns). The inset represents the low-strain region, revealing the differences in elastic behavior. (b) Cooling process from 620 to 240 K over 40 ns (left). MSD evolution by temperature (right) shows lower mobility for materials with high *T*_g_ (red) compared to low *T*_g_ (green). Since all *T*_g_ values are determined based on calibration with the experimental *T*_g_ of a reference system, they represent relative rather than absolute values.

*T*_g_ is determined by plotting the mean square displacement (MSD) against temperature during a cooling cycle^58^ from 620 to 240 K. The MSD value (nm^2^/ns) corresponding to the experimental *T*_g_ of L-F3 system at 87% conversion is set as the reference average atomic mobility. The temperature at which other systems exhibit this reference mobility is identified as their *T*_g_ (Figure 2b and Figure S14), some of which are validated against experimental values. Our analysis, similar to previous observations,^59,60^ demonstrates that values derived from volume-temperature curves are highly sensitive to the fitting procedure. While these methods generally follow a similar trend to MSD results, conventional methods do not quantitatively align with experimental data and exhibit considerable errors. In contrast, the proposed MSD-based method estimates *T*_g_ by directly analyzing the mobility of polymer segments or groups as a function of temperature. This method avoids reliance on secondary properties like volume or density changes and resolves limitations of volume-based calculations, where the nonlinear relationship between estimated *T*_g_ and heating rates complicates accurate estimation at the high heating rates used in simulations.^28^ More data regarding the MSD method, its validation, and the results from the conventional V-T method can be found in SI, Section S2.7.2, along with Figures S13–17. It is important to note that all simulation settings explained in Sections 2.1.1 through 2.1.5 were identical across all systems.

### 2.2 Experimental Investigation

The acrylic resin was synthesized via solution polymerization using the same monomer composition and functionality as the L-F3 prepolymer in simulation. Resin composition was confirmed by ^1^H NMR and molecular weight was characterized via GPC. Hexa­(methoxymethyl)­melamine was used as the cross-linker, with the same acrylic-to-melamine molar ratio in the simulation. A mixture of acrylic and melamine was cast onto a circular silicon mold and cured at 150 °C for different durations (5, 10, 20, and 40 min) to obtain free-standing films of cross-linked acrylic-melamine network. The extent of curing (conversion) was quantified using ATR-FTIR by monitoring the consumption of hydroxyl groups, and further validated by changes in C–H absorption bands. Storage modulus and glass transition temperature were characterized using DMA and DSC, respectively. Detailed information on the materials used, the synthesis process, and the preparation and characterization of free-standing films are provided in SI, Section S1, along with Figures S1–S4 and Tables S1 and S2.

## 3 Results and Discussion

### 3.1 Network Microstructure Analysis

Figure 3 highlights key features of polymer networks as a function of reaction conversion, defined as (eq 1):

$$\mathrm{conversion}\;(\%)=\frac{\mathrm{number}\;\mathrm{of}\;\mathrm{reacted}\;\mathrm{OH}\;\mathrm{and}\;\mathrm{COOH}\;\mathrm{groups}}{\mathrm{total}\;\mathrm{number}\;\mathrm{of}\;\mathrm{OH}\;\mathrm{and}\;\mathrm{COOH}\;\mathrm{groups}}\times 100$$ 1

**Figure 3:**
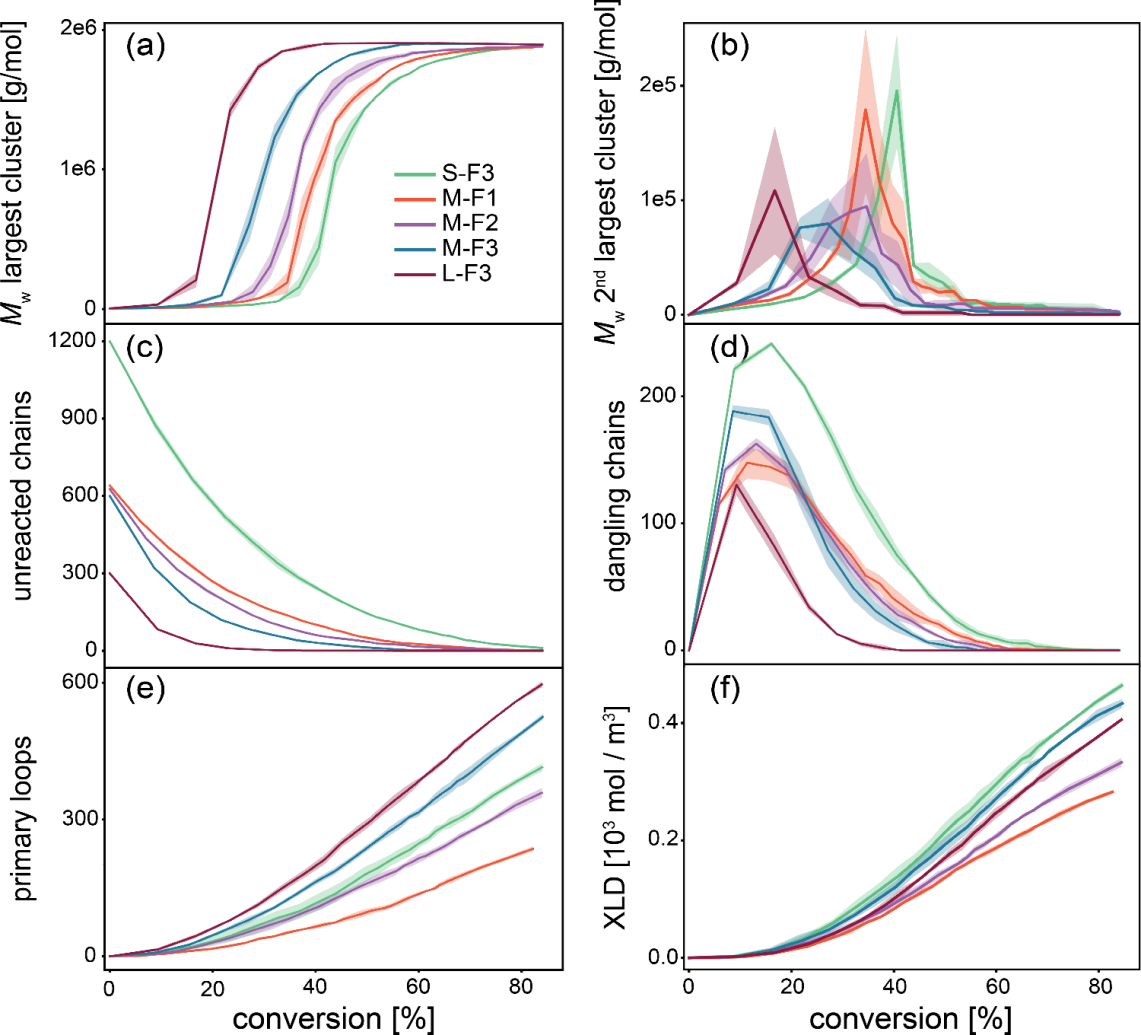
(a) *M*_w_ of the largest cluster as a function of conversion. The standard deviation is shown as a transparent area at the top and bottom of each graph. (b) *M*_w_ of the second largest cluster as a function of conversion. (c) Change in the number of unreacted (free) chains with conversion. (d) Number of dangling chains with conversion. (e) Number of cross-linkers that formed primary loops. (f) Evolution of cross-link density during the reaction.

The molecular weight analysis of the largest cluster (Figure 3a), second largest cluster (Figure 3b), and number of unreacted chains (Figure 3c) aligns with characteristics of step-growth polymerization: (i) high molecular weight clusters form only at high conversions (Figure 3a), and (ii) oligomers are prevalent until near-completion (Figure 3c). The cluster evolution occurs in three stages. Initially, small clusters of two or three chains form, with cluster size increasing sooner as chain length and functionality rise: at 35%, 20%, and 10% conversion for S–F3, M-F3, and L-F3 systems, respectively. In the second stage, cluster size grows rapidly as smaller clusters merge. Finally, molecular weight plateaus as all clusters connect, and further reactions strengthen existing connections in the network rather than forming new clusters. Detailed studies about conversion evolution, and cross-linker reactions are reported in SI Sections S3.1–S3.3.

To further validate our simulations, the gel point for each system is determined using several approaches as shown in Table 2 (the methodologies are detailed in SI Section S3.4). In our simulations, the gel point is identified as the conversion at which the largest cluster merges with the second-largest, which causes a sharp drop in the molecular weight of the second-largest cluster. The peak of this curve marks the gelation point (see Figure 3b). As prepolymer chain length increases, gelation occurs at lower conversions: 42%, 30%, and 20% for S–F3, M-F3, and L-F3 systems, respectively. Longer chains contribute to earlier gelation because their higher number of reactive sites per chain increases the likelihood of forming cross-links that accelerate network formation. The prepolymer functionality has a similar effect, although the chain length has a more pronounced impact. It is worth noting that Flory–Stockmayer and Miller–Macosko equations underestimate the gel point by neglecting the intramolecular reactions,^61^ leading to systematically lower theoretical values compared to our results (see Table 2).

**Table 2:** Gelation Points from Simulation and Theoretical Equations

	*M*_w_ of system	*M*_w_ of 2nd largest cluster	RMW^a^	Flory–Stockmayer	Miller–Macosko
S-F3	42%	41%	41%	28%	33%
**M-F1**	35%	38%	38%	26%	30%
**M-F2**	36%	36%	36%	22%	26%
**M-F3**	30%	29%	29%	18%	20%
**L-F3**	20%	18%	18%	14%	15%

a Reduced molecular weight (see SI Section S3.4).

The number of unreacted chains initially decreases rapidly (Figure 3c). As the reaction progresses, the rate slows down until no unreacted (free) chains remain. This behavior is expected because at the start of the reaction the acrylic chains are sufficiently mobile. This allows them to react easily with melamine. However, as the conversion and degree of cross-linking increase, the mobility of the segments decreases the accessibility of the reactive species. Furthermore, we observe a faster reduction of free chains in systems with longer prepolymers, with the number of free chains reaching zero at approximately 85% conversion for S–F3, 60% conversion for M-F3, and 30% conversion for L-F3. This trend is due to the higher number of functionalities per chain in longer chains (see Figure 1), which increases the likelihood of connecting each chain to the growing network, even though the functionality per unit mass is the same across these three systems. The effect of prepolymer functionality is similar to that of chain length. Nevertheless, as the reaction approaches higher conversions (>70%), the curves for all systems begin to converge, indicating that regardless of the initial chain length or functionality, the highly cross-linked network eventually reaches a state where very few free chains remain.

Dangling chains are considered as a key defect in polymer networks, connecting to the network from one side and therefore not contributing elastically. As shown in Figure 3d, the number of dangling chains initially rises, peaks, and then completely disappears. The peak occurs at nearly the same conversion for all systems. However, the peak heights, representing the maximum number of dangling chains, vary with prepolymer chain length and functionality. Longer prepolymer chains, having higher reactive groups per chain, lead to a lower number of dangling chains during cross-linking because they have a higher chance to react again through their other functionalities and form strands that contribute to network elasticity. The peak also sharpens as the chain length increases, indicative of a faster reduction in dangling chains after the peak. For M-F2 and M-F1 systems, the peak width is similar though the height increases slightly with higher functionality; they present almost the same disappearance rate for dangling chains. It suggests that after the maximum point has been reached, the functionality of the chains is less influential than their length in the removal of the dangling chains.

The formation of loops, i.e., another nonelastic feature, shows a similar trend across all systems (Figure 3e). Loops are intramolecular linkages that reduce the number of elastically effective strands, thereby diminishing the material’s mechanical performance.^20,21,27,62^ Initially, loop formation is slow but accelerates, increasing almost linearly with the reaction progress. This acceleration occurs because the growing network reduces the availability of unconnected cross-linkers and rises the likelihood of cross-linkers reacting with nearby sites on the same chain, enabling chains to form multiple connections with a single cross-linker, which is more likely to happen for longer prepolymers. The highest number of loops is observed in L-F3, where nearly 70% of cross-linkers form loops at 85% conversion. Due to the six reaction sites in HMMM, more complex loops—those involving more than two connections with a single acrylic chain—become more prominent, especially in systems with higher chain lengths and functionality (see SI Section S3.5).

A common conceptual basis in polymer networks is that a cross-link point is a connecting site between several polymer chains in the network; however, the definition of XLD may vary across different simulation studies depending on system complexity and the nature of cross-linking.^13,63–65^ In this paper, XLD was defined as the number of HMMM molecules connecting at least three different acrylic chains divided by the volume of the simulation box. In this way, we exclude the cross-linker molecules which do not contribute to the formation of the 3D network. As shown in Figure 3f, XLD decreases slightly with prepolymer chain length and rises significantly with increasing prepolymer functionality. The former is attributed to the higher likelihood of loop formation in longer prepolymers. Shorter chains, with fewer reactive groups per chain, are less likely to allow HMMM molecules to form multiple connections to the same chain, resulting in a higher XLD. The latter observation is a logical consequence of the increased molar volume of melamine groups in systems with higher functionality acrylics, as we maintained a constant melamine/acrylic ratio across all systems.

### 3.2 Thermomechanical Properties

To explore the correlation between conversion and XLD with the thermo-mechanical properties, we performed simulations to assess two key properties: elastic modulus and *T*_g_. These properties were calculated using atomistic simulation of cross-linked polymers, generated through reverse-mapping methodology as inspired by Backward^48^ and full implementation and modifications are explained in SI Section S2.6. To investigate the structural changes upon reverse mapping, first, we analyze the cluster distributions in the system at both the CG scale and the atomistic level. The results (detailed in SI Section S2.6) demonstrate that the number of clusters were identical in both representations, validating the accurate transfer of bonding information during the reverse-mapping procedure. Then, we analyze the RDFs of different parts of the polymer and cross-linker for (i) the equilibrated CG model, reverse-mapped atomistic model (ii) before and (iii) after extensive equilibrations (over 100 ns at elevated temperatures). As shown in Figure 4, RDFs obtained from the equilibrated atomistic models is in good agreement with the CG model, highlighting the accuracy of the CG force field in capturing general topological features of the network. Upon reverse-mapping, the atoms are placed in the correct positions and system is energy minimized, however, further equilibration at atomistic scale is needed to provide microstructural adjustments. The smoothing of the spikes in RDF upon further equilibration in atomistic scale confirms such local equilibrations. This marks the starting point of simulations for calculation of mechanical and thermal properties.

**Figure 4:**
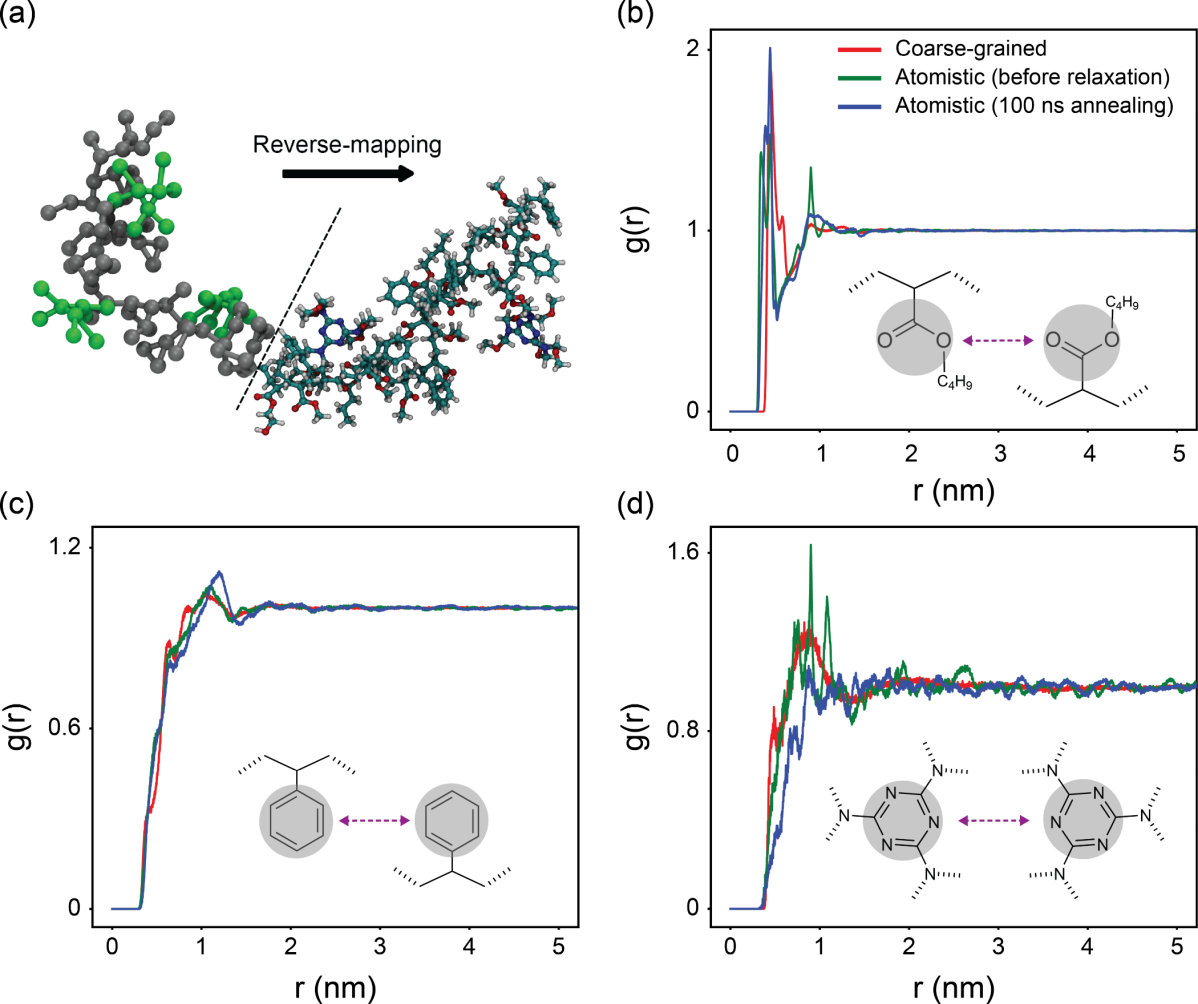
(a) Illustrative representation of the reverse-mapping process transitioning from coarse-grained to atomistic resolution. (b-d) Radial distribution functions, showing structural correlations between: (b) ester groups in prepolymers, (c) benzene groups in prepolymers, and (d) triazole rings in cross-linkers.

The elastic modulus and *T*_g_ of networks are calculated at three different conversions (21%, 55%, and 85%) for all systems and the result of at least three independent samples are plotted in Figure 5. These representative conversions sufficiently capture the evolution of the network structure. The elastic modulus increases as the reaction progresses for all systems, which is consistent with the results obtained from DMA for L-F3 system. The values obtained from the simulation at medium (55%) and high (85%) conversions are approximately 1.76 and 2.0 GPa for the L-F3 system. The experimental results also show an increase in the modulus from 1.47 to 2.0 GPa, in the glassy state, which is in good agreement with the trends and ranges observed in simulations. While MD simulations often overestimate the modulus due to high strain rates, this effect is minimized here since deformation occurred well below simulated *T*_g_, where rate sensitivity is reduced. As a result, the predicted modulus remains consistent with experimental data.^13,57,66–70^ Similar to elastic modulus, the *T*_g_ shows an increasing trend by conversion which is in agreement with the results obtained from DSC at different conversions. The lowest calculated *T*_g_ belongs to S–F3 system, attributed to the late formation of the network and higher chains mobility. In contrast, in the L-F3 system, a rigid cross-linked network result in a higher calculated *T*_g_. In polymer networks with similar chemistry but varying numbers of connectivity, the network topology typically explains differences in thermal and mechanical behavior at different conversions. However, as shown in Figure 5, XLD does not clearly correlate with the elastic modulus or *T*_g_, particularly at low and high conversions, probably due to the network’s topological rearrangements or defects like dangling chains and loops, which cannot be captured by XLD alone.

**Figure 5:**
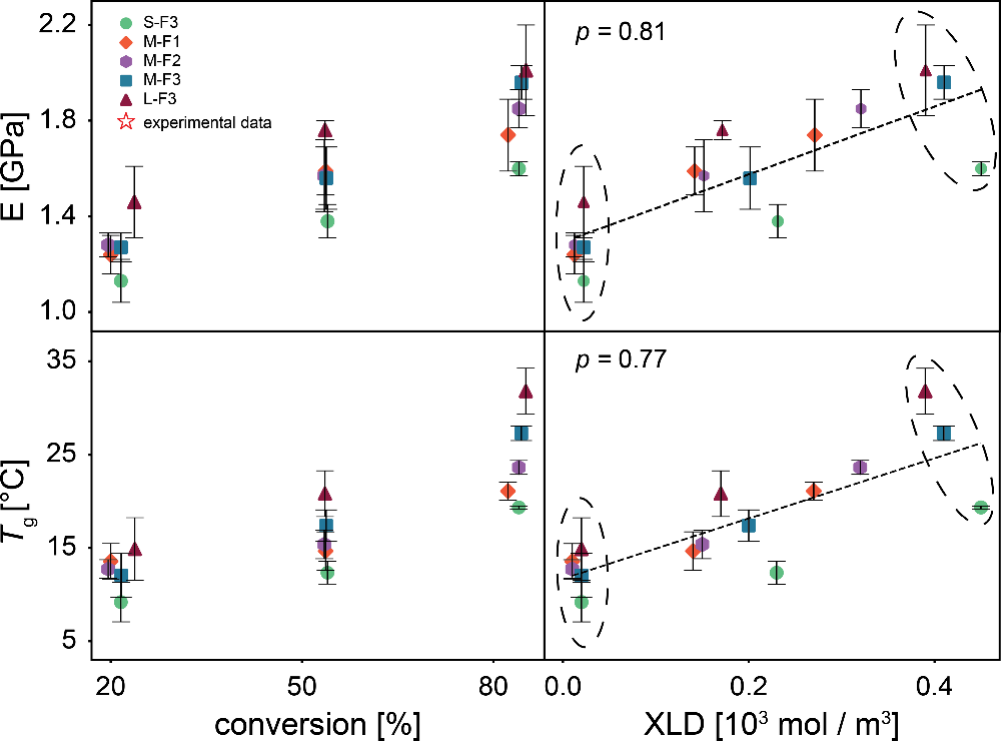
Calculated elastic modulus and *T*_g_ as a function of conversion, XLD. *p* is the Spearman correlation coefficient, and error bars are the standard deviation.

### 3.3 Modifications on XLD

Our approach refines the calculation of cross-link density by addressing the diversity and complexity of effective connections in polymer networks. At its core, we adopt a weighted approach based on the elastic contribution of each cross-link point that includes all the connecting sites contributing to the network. The weighting for each cross-link point, ranging from 2 to 6, is determined by the number of different molecules it connects, with a coefficient equal to this number assigned to it. This consists of three steps of modification:

First, in addition to counting (only) cross-linker molecules with at least three distinct connections to the network as cross-linking points, we need to include points along the prepolymer (acrylic) chains where a connection to the network through three independent paths exists, e.g., see green squares in Figure 6a. Using this counting method, dangling chains and functionalities presented at both the beginning and the end of the chain are excluded, e.g., see red squares in Figure 6a. This adjustment more accurately accounts for the complexity of networks resulting from branched prepolymers, especially for systems with shorter chain lengths, e.g., S–F3 model, with larger number of chain ends. Since these points connect two individual molecules, i.e., a cross-linker to a (acrylic) prepolymer, we assign an elasticity coefficient of two to this type of connection.

**Figure 6:**
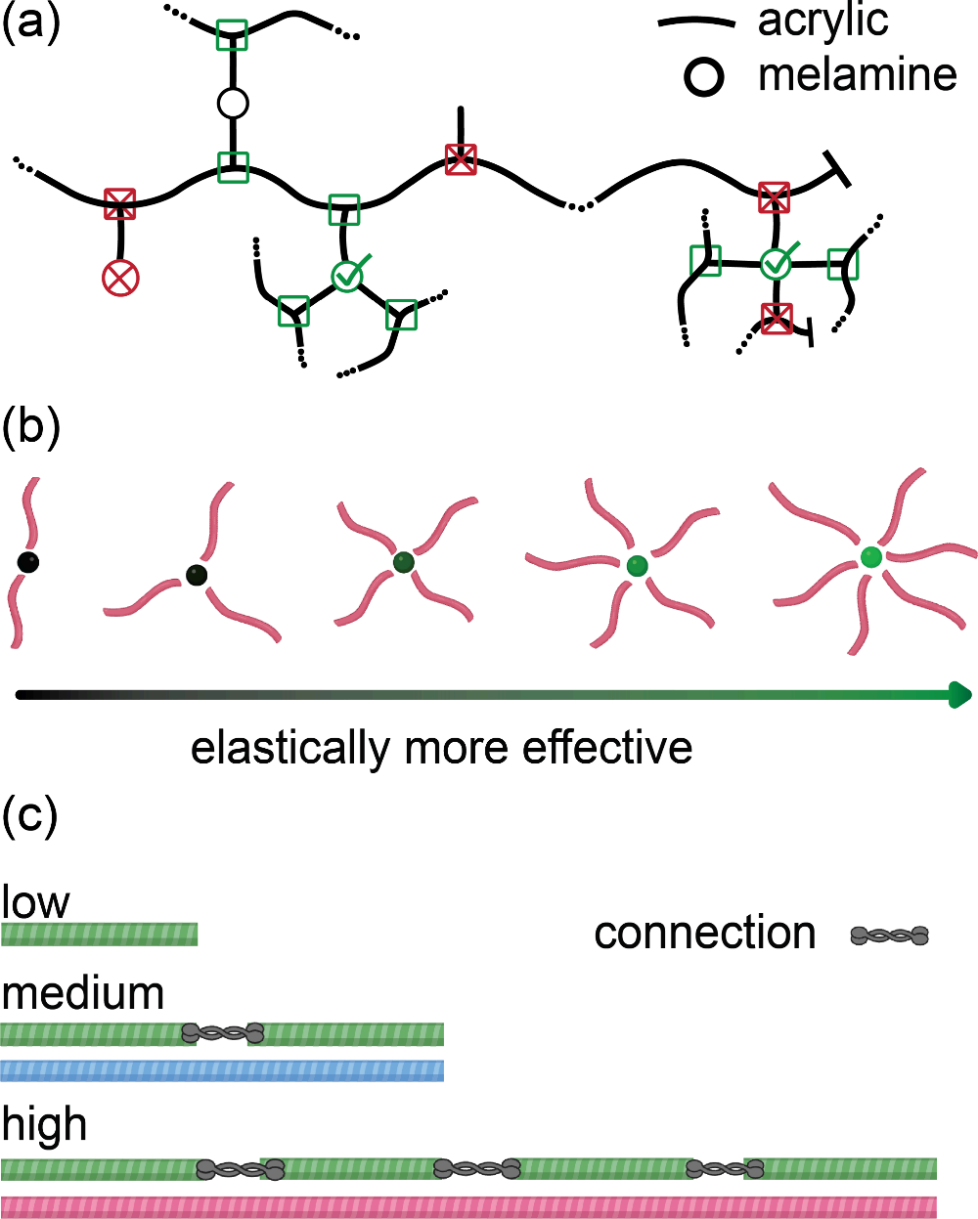
(a) Cross-link points on prepolymer chains. Green squares represent counted cross-link points based on first correction and red squares excluded by this approach (b) Schematic of cross-link points in order of elastic effectiveness. It should be noted that the connections between a chain and a cross-linker are considered as one junction regardless of the number of bonds between them. Even if a melamine forms multiple bonds with the same chain, only distinct chains are considered (c) Connectivity representation for structural units of different sizes.

Second, all cross-linkers are not equally effective in contributing to the network’s elasticity due to variations in the number of individual molecules they connect (Figure 6b). Cross-linkers that connect more distinct chains to the network contribute more effectively by creating additional elastic pathways. Since melamine has 6 functionalities, it can attach to between three and six different chains to be counted as a cross-link point. Therefore, we use weights equal to 3, 4, 5, or 6 for melamine molecules connected to 3, 4, 5, or 6 individual prepolymers, respectively. This correction is particularly significant in systems with highly functional cross-linkers, where it is more likely to have loops and defects in the network that are not elastically effective.

Third, the length of prepolymer chains directly affects their elastic contribution, making it necessary to distinguish between structural units of different sizes. We treat longer chains as composites of multiple smaller chains, i.e., low *M*_w_, connected by linkages, incorporating this effect when counting effective connections. For example, using low *M*_w_ prepolymer as the reference chain, each prepolymer with medium *M*_w_ consists of two reference chains connected by a single “hypothetical” link, and each high *M*_w_ prepolymer consists of four reference chains connected by three “hypothetical” links (see Figure 6c). Since these hypothetical points connect two different molecules, a coefficient of 2 is given to each connection point. This adjustment accounts for the higher intrinsic elasticity of longer prepolymers in the network. To enhance clarity, a detailed step-by-step calculation of XLD^eff^—along with all relevant parameters has been provided for a representative system in Section S3.7 of the SI.

By summing up the additional connections summarized in the above three categories, we modify the concept of XLD and propose XLD^eff^, both in (mol/m^3^ unit). Detailed parameters and step-by-step calculation of XLD^eff^ for a representative case are provided in the SI, Section S3.7, along with Equations SE7, SE8 and Table S11. It is worth mentioning that our approach to modifying cross-link density aligns with the theory of rubber elasticity in characterizing elastically effective strands in polymer networks.^71,72^ In particular, this theory establishes that the elastic modulus is proportional to the density of elastically effective strands, which excludes noncontributing defects such as loops and dangling chains. Additionally, our approach shares conceptual foundations with prior works that emphasize the importance of elastically effective strands, such as the studies by Johnson and Olsen^3,7,20,21,23–26,72^ on loop formation and those by Nourian and Peters^73,74^ on cyclic topologies. However, unlike these studies, which often focus on specific defect types in relatively simple end-linked or low-functionality systems, our XLD^eff^ metric consolidates the collective contributions of all topological features, including network defects and the elastic contribution of each cross-link points, all into a single parameter.

The XLD^eff^ is calculated during the reaction and plotted against the elastic modulus in Figure 7. By fitting a line to these data points, we observe a linear trend that can predict the elastic modulus for systems with similar chemistry without the need for additional experiments. The high value of Spearman’s coefficient for this data set (*p* = 0.97) shows a strong linear correlation between the elastic modulus and XLD^eff^. To assess whether real experimental systems exhibit similar structure–property trends as those predicted by our simulation-derived metric, we assume that the XLD^eff^ values of the experimental samples at the calculated conversions (see Table S2) could be directly applied to the L-F3 system. This enabled the placement of experimental data points on XLD^eff^–property plots at corresponding conversion levels (Figure 7). Notably, this assumption was used only for qualitative comparison and was not involved in computing or interpreting mechanical properties or *T*_g_ values in the simulations. XLD^eff^ shows a strong correlation with calculated *T*_g_ (*p* = 0.95), providing a good prediction of thermal properties. Therefore, in contrast to conversion and XLD, XLD^eff^ gives a more comprehensive representation for microstructural features that affect the thermomechanical performance of the network. It is worth noting that we do not expect a perfect rank correlation due to the influence of nonbonded interactions, which arise from changes in molecular structure during curing (e.g., conversion of polar to nonpolar groups).

**Figure 7:**
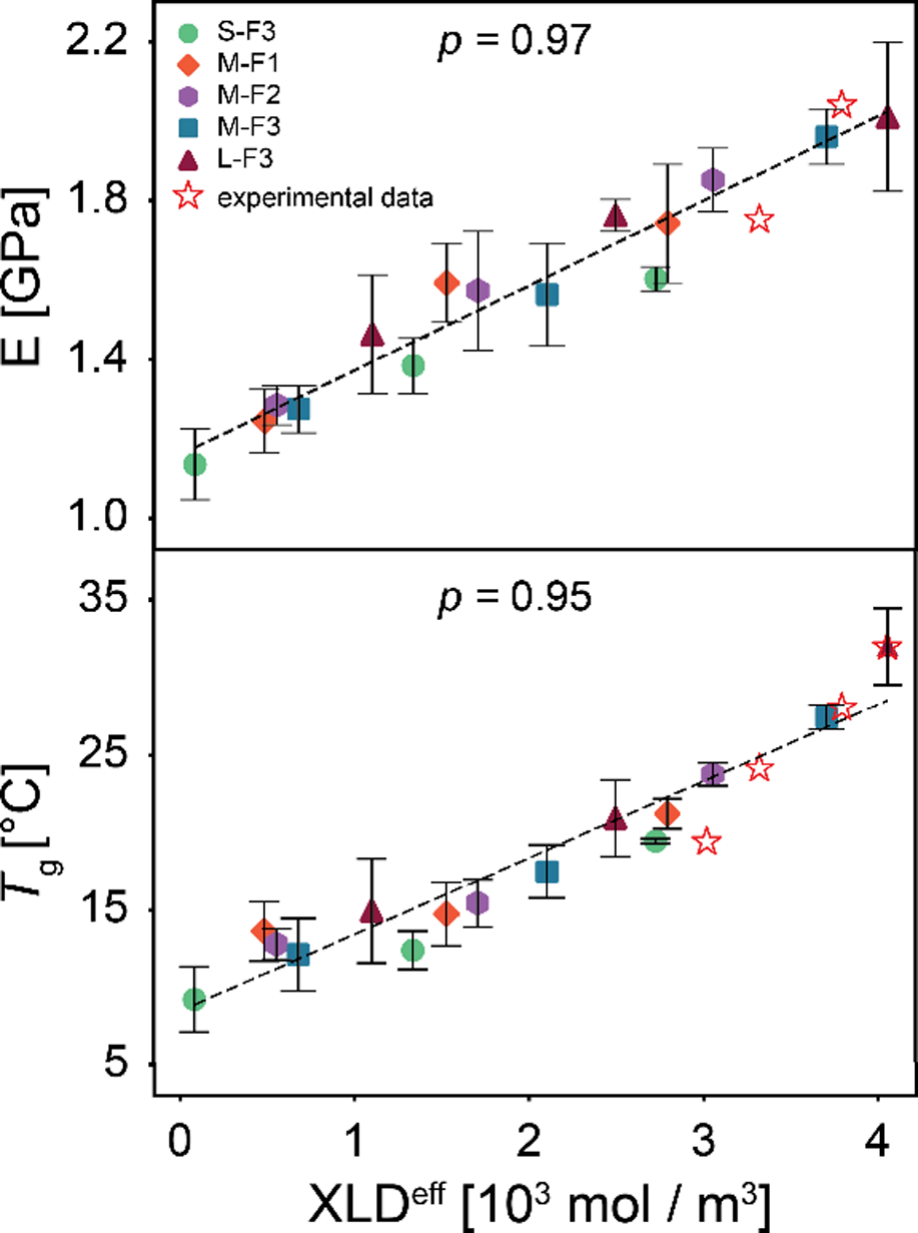
Calculated elastic modulus and *T*_g_ as a function of XLD^eff^. *p* is the Spearman correlation coefficient, and error bars are the standard deviation.

It is important to note that, in the glassy regime and under high strain rates, typical for atomistic simulations, nonbonded interactions and caging effects contribute significantly to the elastic response, even in the absence of network connectivity. As a result, the elastic modulus does not drop to zero at XLD or XLD^eff^ = 0, as uncross-linked systems still exhibit nonzero stiffness due to chain entanglement and nonbonded interactions. Nevertheless, all systems examined in this study share the similar underlying chemistry, including monomer type, repeating unit pattern, and cross-linking conditions. Therefore, the contribution of nonbonded interactions is expected to be approximately constant across different systems. The primary factor distinguishing the systems is the network microstructure, particularly the connectivity of cross-linkers and polymer chains, which are effectively captured by XLD^eff^. Therefore, although our study primarily focuses on thermoset systems, the definition of XLD^eff^ is based on general structural features common to various polymer networks, including cross-linker functionality, topological defects (e.g., loops, dangling chains), and the elastic effectiveness of cross-link points. These features are also relevant in other network-forming systems, such as rubbers, and hydrogels, where the network topology and polymer chain connectivity remain critical in determining thermomechanical response. Therefore, the insights gained from our study are applicable to a broader range of polymer networks.

All in all, our simulations provide a clear picture of the curing process of these networks, which potentially informs the formulation optimization. For instance, there is common knowledge that high-solid acrylic resins need to reach very high conversions to give acceptable thermomechanical properties^75^ and our results unravel the reasons behind this. High-solid resins (similar to our short chain, S–F3) form fewer loops compared to longer acrylic resins (e.g., L-F3), and therefore, a higher proportion of cross-linkers create connections that are elastically more effective by bonding to more distinct polymer chains. However, the higher ratio of the end-group functionalities in the branches in each prepolymer chain that cannot act as a cross-link point, combined with the lower elasticity of the shorter chains themselves lead to lower elastic networks in medium conversion ranges so that comparable mechanical properties with networks made from conventional (longer chain) resins requires reaching to very high conversions. Additionally, since their gelation point occurs at higher conversions, the acceptable conversion range at which the properties are reached is narrower. This explains the reason behind the crucial role of controlling reaction conditions for these systems to enable reaching acceptable performance.

## Conclusions

This study provides a deeper understanding of structure–property relationships for complex polymer networks, which led to the introduction of XLD^eff^, a single parameter for the assessment of thermomechanical properties, through three modification steps. XLD^eff^ builds on this idea by first identifying cross-linking points that are effectively integrated into the network, and then weighting them based on their elastic contribution. This allows us to illustrate the relationship between network structure and thermomechanical properties by defining a single metric. The correction to XLD, which accounts for the number of distinct chains attached to each melamine (step two), shows its impact particularly at higher conversions, when all melamine types (ranging from those attached to 3 to 6 distinct chains) have had time to form. In contrast, the adjustment for effective cross-linking through branch points (step one) shows its impact starting at midlevel conversions as the network begins forming, significantly improving its correlation with E and *T*_g_. XLD^eff^ is more than just a metric for correlating mechanical and thermal properties with network microstructure. It provides valuable insights into often-overlooked microstructural features that influence macroscopic behavior, such as dangling chains, loops, and variations in cross-linker connectivity, as well as their relationship to formulation choices like the molecular weight and functionality of prepolymers. Additionally, XLD^eff^ offers a computational framework for in-silico design of polymer networks with tailored properties by accounting for controllable microstructural parameters in formulation design. This makes it a significant advancement for both theoretical research and practical applications in the design of complex polymer networks.

## Supplementary Material



## Data Availability

To promote Open Science practices, all code and files used to perform our simulations and analysis are available on the GitHub page of our group: (https://github.com/HMakkiMD/PolymerNetwork).

## Notes

Detailed information on the parametrization of structures, reaction procedures, system size analysis, reverse mapping to the atomic scale, simulating the tensile test, further analysis on network microstructure, and *T*_g_ determination (PDF)
