# Supplementary material for: Effective Cross-Link Density as a Metric for Structure–Property Relationships in Complex Polymer Networks: Insights from Acrylic Melamine Systems
Source: ACS Appl Polym Mater. 2025 Jul 17;7(14):9034–44. doi: 10.1021/acsapm.5c01155 (PMC12305489; doi:10.1021/acsapm.5c01155)
Supplement: Supplementary file 1 [file ap5c01155_si_001.pdf]

Supporting Information For:

Effective Crosslink Density as a Metric for Structure–Property Relationships in Complex Polymer Networks: Insights from Acrylic Melamine Systems

Amirhossein Gooranorimi†, Seyyed Mohammad Mousavifard†, Mohsen Mohseni†, Hossein Yahyaei†, Hesam Makki‡*

† Department of Polymer and Color Engineering, Amirkabir University of Technology, 424 Hafez Avenue, Tehran 15875-4413, Iran

‡ Department of Chemistry and Materials Innovation Factory, University of Liverpool, Liverpool L69 7ZD, U.K.

Corresponding author: Hesam Makki (h.makki@liverpool.ac.uk)

S1. Experimental Method

S1.1. Materials

An acrylic resin containing the same molar ratios of styrene, methyl methacrylate (MMA), 2-hydroxyethyl acrylate (HEA), butyl acrylate (BA), and 2% wt. acrylic acid monomers (Hydroxyl content = 3.3 %) was synthesized in solution polymerization and methoxy propyl acetate (MPA) as the solvent. This resin is similar to the prepolymer of L-F3 model in simulation. CYMEL 303, a highly methylated monomeric melamine (supplied from Allnex), containing 98% hexa(methoxymethyl)melamine (HMMM) with M_w = 354 g/mol was used as cross-linker. A blend of MPA and xylene was prepared as solvent for adjusting the viscosity of the resin mixture during application. All monomers and solvents were purchased from Sigma-Aldrich.

S1.2. Resin characterization

NMR

NMR spectroscopy was utilized to confirm the chemical structure of the synthesized acrylic resin and to determine the composition of the copolymer. The resin samples were placed in a vacuum oven at 50°C for 6 hours to remove any volatile components. Then, the NMR spectra of resins and monomer mixtures (before synthesis) were recorded on a Bruker (Avance-400) NMR spectrometer operating at 400 MHz for ^{1}H-NMR. The samples were dissolved in $CDCl_3$ and a total of 16 scans were accumulated for each spectrum to ensure a good signal-to-noise ratio. The ^{1}H-NMR spectrum showed characteristic peaks corresponding to the protons of styrene, MMA, HEA, and BA, confirming the presence of monomer units within the copolymer structure, indicating successful copolymerization (Figure S1). The integration of peaks allowed for the determination of the monomer composition within the copolymer, showing the same ratios of the monomers in the feed of the reactor (Table S1).

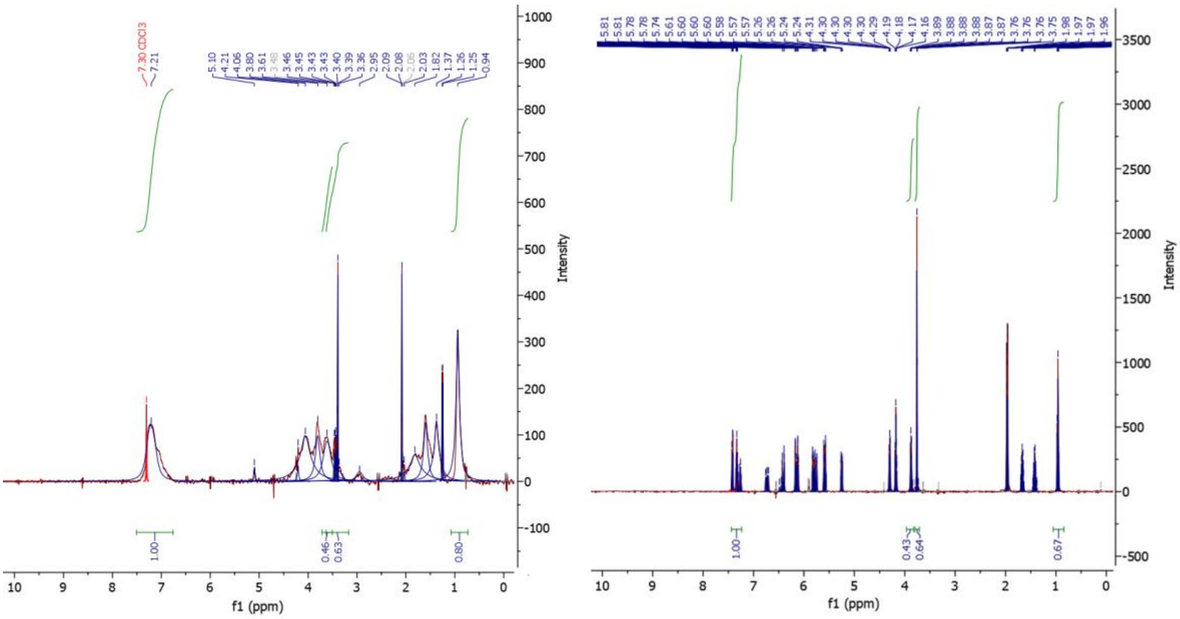

Figure S1. 1H-NMR spectra from the synthesized resin (left) and the mixture of monomers in the feed (right).

Table S1. Characteristic peak of styrene monomer was taken as the reference and the ratio of the peak's integration for each monomer to the reference was calculated in both monomers' mixture before the polymerization (in feed) and the synthesized resin (in resin).

peak ratio	BA / styrene	HEA / styrene	MMA / styrene
in feed	0.67	0.43	0.64
in resin	0.80	0.46	0.63

Gel Permeation Chromatography (GPC) was employed to determine the molecular weight of the synthesized acrylic resin. The analysis was performed using an Agilent-1100 series GPC system and THF as the solvent. GPC analysis revealed the molecular weight of the polymer, with an M_n of 5818 g/mol.

S1.3. Sample preparation

A mixture consisting of a 1:2.8 molar ratio of acrylic to HMMM, which corresponds to a 1:1.2 molar ratio of their functional groups, was completely stirred with about 5% wt. solvent for 2 h to achieve a uniform solution with 60 w% solid content. Then, 1 g of the mixture was cast on a circular silicon mold with a 3 cm diameter and cured in an air oven at 150 °C to obtain free films with a thickness of 300±20 µm (Figure S2).

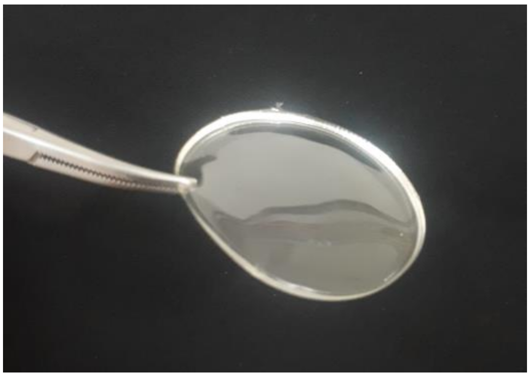

Figure S2. Free-standing film of a cross-linked acrylic-melamine network

S1.4. Film characterization

ATR-FTIR

ATR-FTIR spectroscopy was performed to determine the extent of curing, i.e., reaction conversion, of the films based on the presence of hydroxyl functional groups. The ATR-FTIR spectra were recorded using a Bruker FTIR spectrometer (Germany) equipped with an ATR accessory. The spectra were acquired in the range of 4000-400 cm^{-1} with a resolution of 1 cm^{-1}. The resulting spectra of the samples confirmed the presence of ester, hydroxyl, and aromatic groups, consistent with the expected chemical structure of the synthesized acrylic resin. Moreover, the ratio of the area calculation of the O-H stretch absorption band observed around 3200-3600 cm^{-1} (indicating the presence of unreacted hydroxyl groups from HEA) to the strong C=O stretch absorption band observed around 1730 cm^{-1} (indicating the presence of ester carbonyl groups of monomers) in cured and uncured mixtures can reveal the conversion of the cross-linking process. In addition, the suppression of C-H stretches absorption band at around 2900 cm^{-1} can be interpreted as the consumption of the methoxy groups of HMMM during the cross-linking and methanol evaporation from the coating indicating another approach for reaction conversion calculation. The calculated conversions at different curing times for both approaches are summarized in Table S2. Accordingly, the results show almost the same values and trends for both approaches indicating a rapid increase in conversion at the beginning of the curing, reaching about 86.5% conversion for overbaked coatings. We use the conversions calculated based on OH groups consumption in this study as the CH absorption band may also be affected by self-condensation of the melamines in addition to the cross-linking reactions. The ATR-FTIR spectra for unreacted and samples taken out after 5, 10, 20, and 40 minutes from the oven are illustrated in Figure S3.

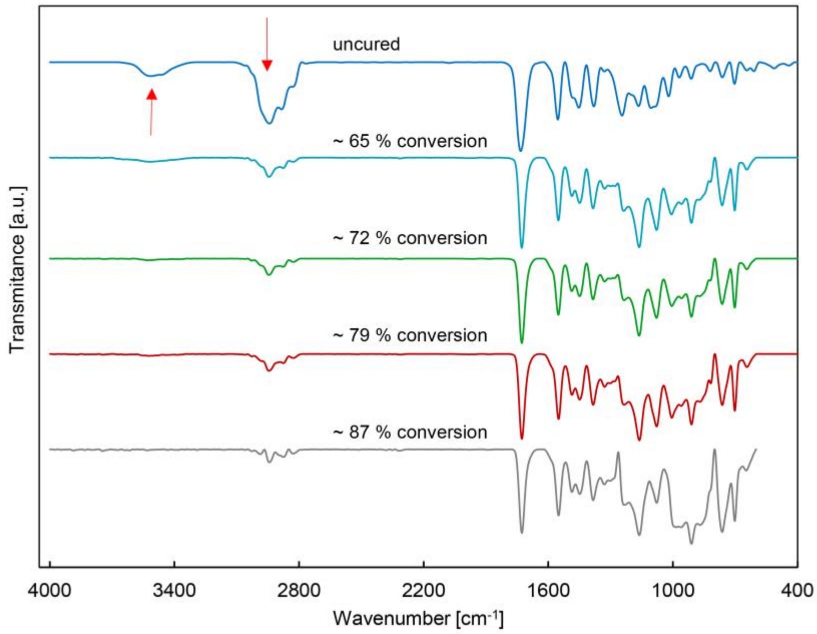

Figure S3. ATR-FTIR spectra for uncured and cured samples. The conversions of 65%, 72%, 79%, and 87% are associated with samples taken from the oven after 5, 10, 20, and 40 minutes, respectively.

Table S2. The conversion of the cured coatings at various curing times calculated based on ATR-FTIR results.

curing time [min]	calculated conversion [%] based on O-H	calculated conversion [%] based on C-H
5	65	63
10	72	70
20	79	76
40	87	83

DMTA

Dynamic mechanical thermal analysis (DMTA Tritec 2000, England) was performed in the tension mode to study the mechanical behavior of free films. The analysis was performed in the temperature range 0 to 125 °C with 5 °C / min heating rate and 1 Hz frequency. The dimensions of the samples were 8 × 6 mm with a thickness of around 0.3 mm.

The samples with different conversions (72% and 79%) were analyzed (Figure S4). Regardless of the close conversion rates, at temperatures below the T_g, the elastic moduli of these samples are markedly different. This increase in strength, observed below T_g, clearly demonstrates improved mechanical properties resulting from extended curing. However, at temperatures higher than T_g, both samples show similar storage modulus. We believe that at these high temperatures, the device was not able to accurately distinguish between the two samples due to the softening and much lower stiffness of the samples.

DSC

DSC measurements were performed by Netzsch machine (Germany) for around 10 mg samples through a 10 ° C / min heating rate. Figure S4 left shows the second heating cycle from -50 to 90 ° C. As shown, T_g shifts to higher temperatures as the conversion increases. This indicates that increased cross-linking between polymer chains restricts their mobility, and a higher temperature needs to reach the same level of dynamics. This principle has been used to estimate calculated T_g from simulations. The values for T_g read 19.3, 24, 27.9, 31.8 °C with increasing the conversion from 65% to 87%.

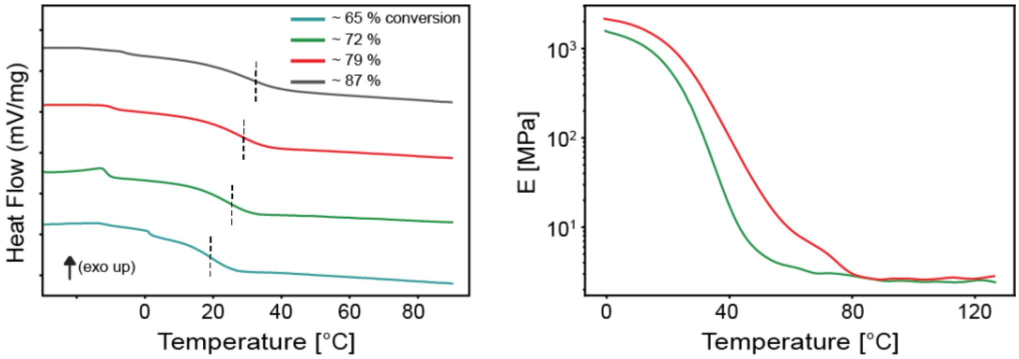

Figure S4. Thermal and mechanical characterization of samples at different conversion levels. Left: DSC curves showing heat flow as a function of temperature. Right: DMA curves showing the storage modulus versus temperature.

S2. Simulation Method

S2.1. All-atom MD simulation

To achieve a reliable coarse-grained (CG) model, atomistic simulations are essential for mapping to CG scale. Additionally, the atomistic models of the constituent elements of polymer networks are necessary for reverse-mapping from CG to atomistic structures after cross-linking reactions to further analyze the thermo-mechanical properties of cross-linked systems. To this end, initial structures of acrylic resin (ACR) and hexa(methoxymethyl)melamine (HMMM), were optimized using Density Functional Theory calculations employing B3LYP/6-31G*. This optimization ensured the molecular geometries were at their lowest energy, which is essential for accurately determining atomic interaction parameters. Partial charge densities were calculated using ESP in Gaussian 16 software, while Lennard-Jones and bonded interaction parameters were taken from the OPLS-AA force field.[1]

Using the GROMACS 2022 package, separate boxes containing 50 randomly inserted ACR (atomistic structure of M-F3 model) and HMMM molecules were created. After energy minimization, the boxes were subjected to high pressure and temperature (500 bar, 500 K) to facilitate packing. Periodic boundary conditions were applied in all three dimensions to mimic an infinite system. Subsequently, NPT simulations were performed for 100 ns to relax the molecules at 300 K and 1 bar, using a V-rescale[2] thermostat and a C-rescale[3] barostat, with a 2 fs time step.

S2.2. CG parametrization

S.2.2.1 Non-bonded parameters

After achieving equilibrium in the atomistic simulations, the structures were mapped to the CG scale based on Martini3 methodology.[4] The mapping includes defining each CG bead to represent a group of atoms based on Martini3 force field instruction. The structures and bead types are illustrated in Figure S5. Non-bonded interactions in the CG model were described by Lennard-Jones 12-6 potential energy functions. The values of σ and ε depend on the type assigned to each bead and were taken from Martini3 force field.

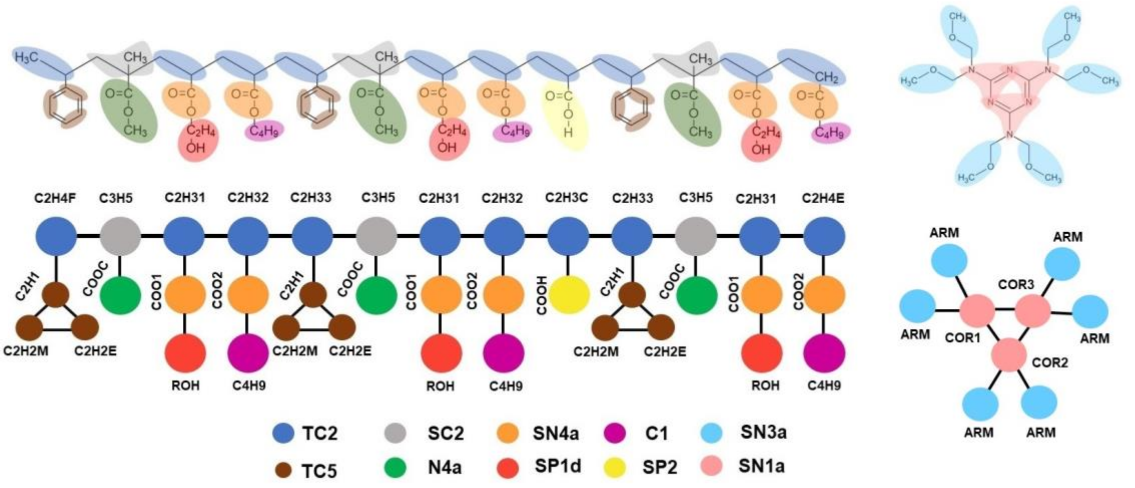

Figure S5. Atomistic and coarse-grained structures for acrylic and melamine based on Martini 3. The colors indicate the type of beads. The name assigned to each bead is also placed next to it.

S2.2.2 Bonded parameters

Next, the bonds, angles, and dihedral potentials connecting the center of volume of the atoms in each bead were averaged from the last 5 ns of atomistic simulation trajectories. Equations SE1-5 were used to calculate the potential values by converting the distribution of bonded parameters into a probability function. Then, the CG potentials were fitted to these equations, determining CG bonded parameters. Tables S3-5 present the equilibrium values and force constants for all bonded interactions.

$$U_{bond} = \frac{1}{2}K_{bond}(l-l_0)^2 \qquad \text{(SE1)}$$

$$U_{angle} = \frac{1}{2}K_{angle}(\cos\theta - \cos\theta_0)^2 \qquad \text{(SE2)}$$

$$U_{angle} = \frac{1}{2}K_{angle}\frac{(\cos\theta - \cos\theta_0)^2}{\sin^2\theta} \qquad \text{(SE3)}$$

$$V_d(\phi_{ijkl}) = K_\phi(1+\cos(n\phi-\phi_s)) \qquad \text{(SE4)}$$

$$V_{CBT}(\theta_{i-1},\theta_i,\phi_i) = k_\phi \sin^3\theta_{i-1} \sin^3\theta_i \sum_{n=0}^{4} a_n \cos^n\phi_i \qquad \text{(SE5)}$$

Table S3. Bond type parameters at CG level.

bond	l_0 [nm]	k_{bond} [kJ mol^{-1} nm^{-2}]	bond	l_0 [nm]	k_{bond} [kJ mol^{-1} nm^{-2}]
C2H1 - C2H4F	0.273	16000	C2H32 - COO2	0.24	7050
C2H33 - C2H1	0.273	12000	C2H4E - COO2	0.243	36900
C2H4F - C3H5	0.342	3640	COO1 - ROH	0.275	13200
C2H33 - C3H5	0.325	3510	C4H9 - COO2	0.389	3760
C3H5 - C2H31	0.258	16200	C2H3C - COOH	0.276	10800
C3H5 - COOC	0.374	13900	C2H1 - C2H2M	0.26	1000000*
C2H31 - C2H32	0.267	8750	C2H1 - C2H2E	0.304	1000000*
C2H32 - C2H33	0.253	8750	C2H2M - C2H2E	0.293	1000000*
C2H31 - C2H4E	0.250	7600	COR1 - ARM	0.351	5650
C2H31 - COO1	0.253	31000	COR1 - COR2	0.233	1000000*

* Constraints

Table S4. Angle type parameters at CG level.

angle	type	θ_0 [deg]	k_{angle} [kJ mol^{-1} rad^{-2}]
C2H1- C2H4F- C3H5	2	75	140
C2H1 - C2H33 - C3H5	2	77	100
C2H4F- C3H5 - COOC	2	80	100
C2H33 - C3H5 - COOC	2	85	35
C2H4F- C3H5 - C2H31	10	107	30
C2H33 - C3H5 - C2H31	10	105	25
COOC - C3H5 - C2H31	2	80	165
C3H5 - C2H31 - C2H32	10	140	30
C3H5 - C2H31 - C2H4E	10	148	52
C3H5 - C2H31 - COO1	2	121	30
C2H31 - COO1 - ROH	2	141	60
COO1 - C2H31 - C2H32	2	74	270
COO1 - C2H31 - C2H4E	2	87	366
C2H31 - C2H32 - COO2	2	115	119
C2H31 - C2H4E - COO2	2	131	40
C2H31 - C2H32 - C2H33	2	151	38
C2H32 - COO2 - C4H9	2	134	70
C2H4E - COO2 - C4H9	2	138	90
C2H32 - C2H33 - C2H1	2	112	97
C2H32 - C2H33 - C3H5	2	137	20
COO2 - C2H32 - C2H33	2	76	162
C2H32 - C2H3C - COOH	2	113	126
COOH - C2H3C - C2H33	2	77	289
C2H32 - C2H3C - C2H33	2	154	44
C2H4F- C3H5 - COOC	2	80	100
ARM - COR1 - ARM	2	75.6	150

Table S5. Dihedral type parameters at CG level.

dihedral	type	ξ_0 [deg]	k_ξ [kJ mol^{-1} rad]
C2H4F - C3H5 - C2H31 - COOC	2	90	7
C2H1 - C2H2M - C2H2E - C2H4F	2	0	110
C3H5 - C2H31 - C2H32 - COO1	2	120	4

dihedral	type	ϕ_s [deg]	k_ϕ [kJ mol^{-1}]
C3H5 - C2H31 - C2H32 - C2H33	1	100	1.5

This bead-typing methodology and parametrization served as a foundational template for constructing structure files of other systems. Given the repeating nature of these polymer units across systems, the bead-typing strategy was consistently applied to other CG structures. The full structures of S-F3, M-F3, and L-F3 models are presented in Figure S6.

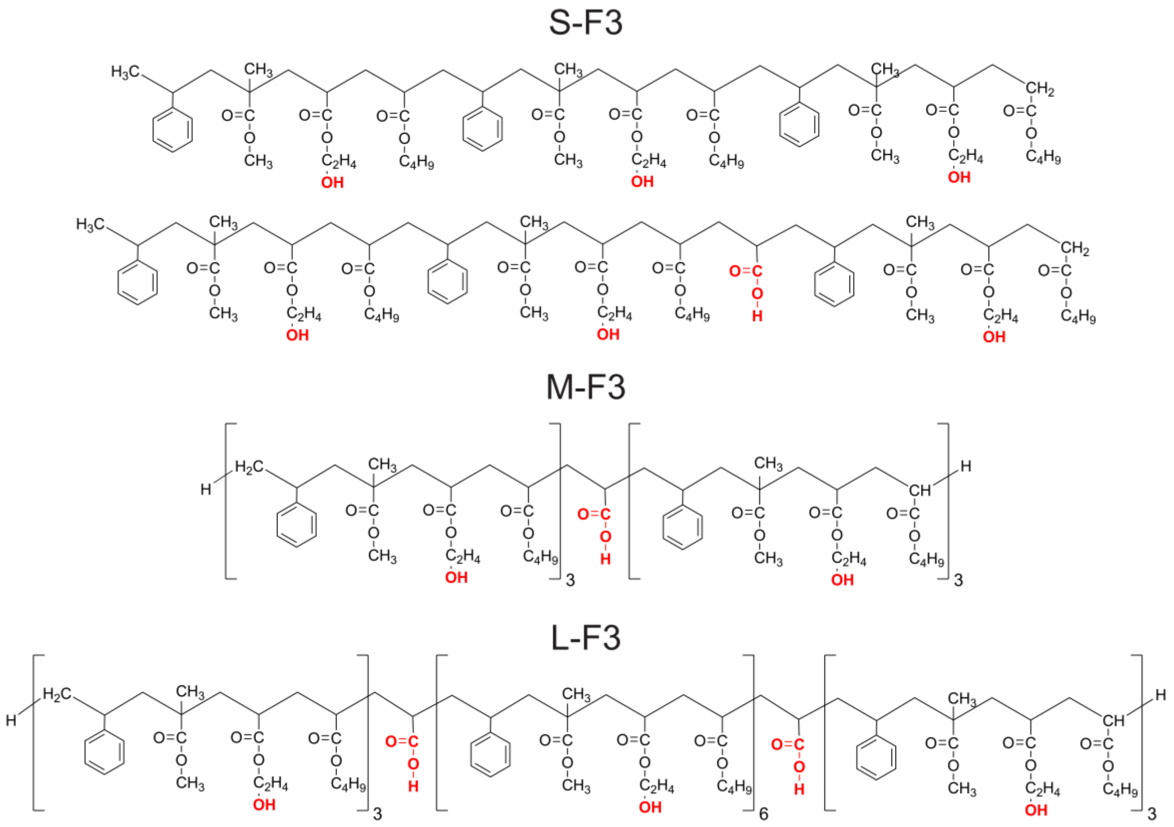

Figure S6. Chemical structures of S-F3, M-F3, and L-F3 prepolymers, with reactive groups highlighted in red.

S2.3. Validation of CG parameters

CG simulations were performed on separate boxes containing 50 molecules of ACR (M-F3 model) and HMMM for 100 ns under NPT condition with a time step of 10 fs using a V-rescale thermostat and C-rescale barostat. The calculations of van der Waals interactions utilized a cutoff method, with a cutoff distance set at 1.1 nanometers, following the Martini force field recommendations. Additionally, the accuracy of the force field for acrylate structures was further verified by performing a simulation under the same conditions on a polymethyl methacrylate (PMMA) with available experimental data.[5] The comparison of key properties, such as radius of gyration, density, and end-to-end distance from both the atomistic and CG simulations against experimental data shows a good agreement that validates proper parameterization of these structures (Table S6).

Table S6. Comparison of densities, radii of gyration, and end-to-end distance. Values are calculated at 300 K. The numbers in the bracket are the difference from the AA simulations.

	Density [kg/m^3]			Radius of gyration [nm]			End-to-end distance [nm]		
	**AA**	**CG**	**Experimental**	**AA**	**CG**	**Experimental**	**AA**	**CG**	**Experimental**
**Acrylic**	1.106	1.165 [5%]	-	1.145	1.126 [1.5%]	-	2.760	2.782 [0.8%]	-
**PMMA**	1.143	-	1.15-1.19[5]	0.97	-	1.10[5]	-	-	-
**HMMM**	1.144	1.207 [5%]	1.18-1.2	0.402	0.389 [3.2%]	-	0.649	0.631 [2.8%]	-

S2.4. Cross-linking procedure

Before starting the reactions, acrylic and melamine molecules were randomly inserted into a simulation box. Following energy minimization and box packing, the system was relaxed for 150 ns under NPT conditions (time step=10 fs, T=300 K, P=1 bar). To ensure the system reached

equilibrium, the autocorrelation function (ACF) of the polymer's end-to-end distance was evaluated during the simulation. ACF provides information about how quickly the system becomes independent of its initial configuration and reaches a state where its properties fluctuate around a stable mean. In molecular simulations, the reduction of ACF to zero indicates that the time-related variation has decreased to the point where no significant correlation remains. As shown in Figure S7, the system reached equilibrium after about 30 ns.

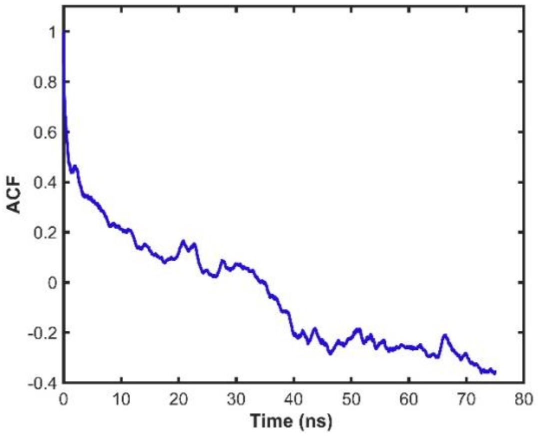

Figure S7. End-to-end distance autocorrelation function over simulation time.

In this study, we used PolySMart[6] to simulate the reactions between functional groups and mimic the formation of network. PolySMart is able to model reaction dynamics based on the pre-determined cut-off radii and the reaction probability for different functionalities. Before the reaction process can be started, for each molecule the reactive beads contributing to cross-linking must be defined. These beads are the reaction sites that new bonds will form during the reaction process. The cut-off radius was set to ± 5% of the Lennard Jones radii of the reacting beads. The reaction probabilities for COOH and OH were set to be equal, and the self-condensation of HMMM was set to 2% of the acrylic-melamine reaction.[7] When a reaction has occurred, the topological information of the reacting beads is updated; this includes new bond generation and changes in the existing bonds affected by the reaction. When the reaction occurs between acrylic and melamine, the bead type of ROH bead changes from *SP1d* to *TC2*, the bead type of COOH bead changes from *SP2* to *TN6a*, and the bead type of ARM bead changes from *SN3a* to *TN3a*. For self-condensation of melamine, the bead type of ARM beads changes to *TN1*. Bonded parameters after cross-linking are provided in Tables S7-10.

Each reaction cycle contains an energy minimization followed by a 50 ps relaxation with 1 fs time step, and then 1 ns relaxation with 5 fs time step to relax the new configurations formed during the reaction. This process iterates until the desired level of conversion is reached. As a result, the structure and topology files of each step are available for subsequent analyses which enables us to have an in-depth exploration of the reaction mechanism at the molecular level.

The cross-linking reaction was performed on three separate samples. The average results from these simulations, along with the standard deviation for each parameter, are reported to ensure consistency. Moreover, all simulation settings were identical across the studied systems.

Table S7. New bond parameters formed after cross-linking.

bond	l_0 [nm]	k_{bond} [kJ mol^{-1} nm^{-2}]
1ROH - 1ARM	0.28	13000
1COOH - 1ARM	0.22	63000
1ARM - 1ARM	0.064	90000

Table S8. New bond parameters formed after cross-linking.

angle	type	θ_0 [deg]	k_{angle} [kJ mol^{-1} rad^{-2}]
COO1 - 1ROH - 1ARM	2	111	75
1ROH - 1ARM - COR	2	132	50
C2H3 - 1COOH - 1ARM	2	116	110
1COOH - 1ARM - COR	2	159	150
COO - 1ARM - 1ARM	2	145	450

Table S9. Change in the existing bond parameters after cross-linking.

bond	l_0 [nm]	k_{bond} [kJ mol^{-1} nm^{-2}]
1COOH - C2H3	0.256	13100
1ROH - COO1	0.262	17000
1ARM [TN3a] - COR	0.28	90000
COR - 1ARM [TN1]	0.229	54500

Table S10. Change in the existing angle parameters after cross-linking.

angle	type	θ_0 [deg]	k_{angle} [kJ mol^{-1} rad^{-2}]
1ARM [TN3a] - COR - 1ARM [TN3a]	2	63	3000
1ARM [TN3a] - COR - ARM [TN3a]	2	67	600
1ARM [TN1] - COR - ARM	2	64	600
1ARM [TN1] - COR - 1ARM [TN1]	2	65	3000
1ARM [TN1] - COR - 1ARM [TN3a]	2	68	600

S.2.5. System size analysis

System size analysis was performed to ensure the results were independent of the number of particles in the simulation box. Initially, the conversion rate for each reactive group was calculated across four systems containing 17320, 34640, 51960 and 69280 beads. As shown in Figure S8, the results do not show a significant difference.

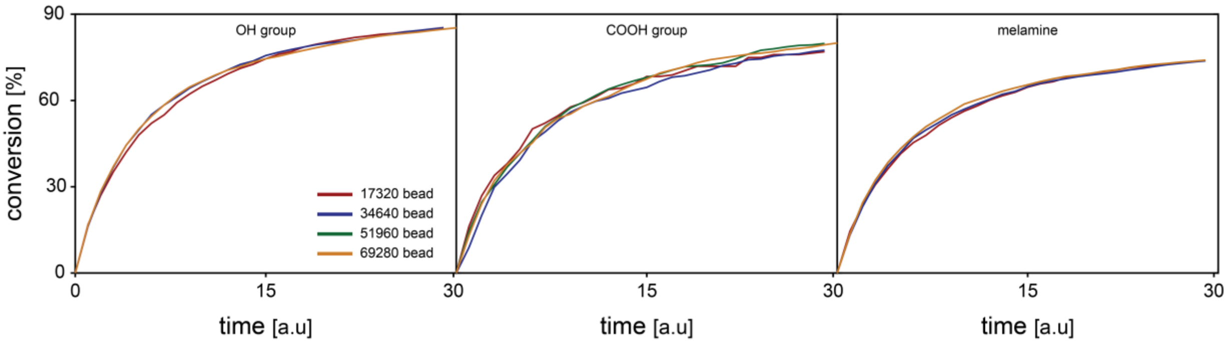

Figure S8. Conversion as a function of reaction time for: (left) hydroxyl group (middle) carboxyl group (right) melamine. The reaction time is presented in arbitrary time units due to the use of relative (and not absolute) reaction rates.

The mobility of the materials was then measured using MSD, along with the cross-link density. These results, illustrated in Figure S9, also show minimal differences across the various system sizes. Despite the similarities in the properties, the system containing 51960 beads was selected for subsequent simulations. This choice balances the need for computational efficiency with the desired accuracy.

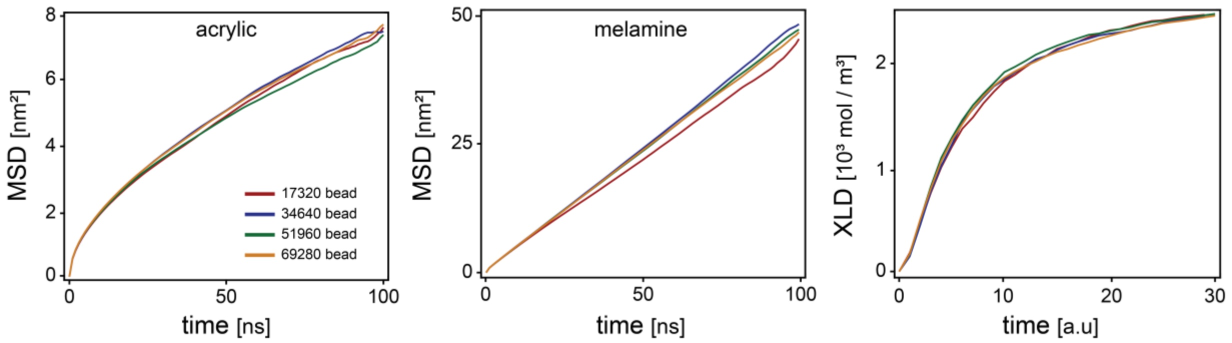

Figure S9. MSD vs. time for: (left) acrylic (middle) melamine. (right) Cross-link density vs. time

S2.6. Reverse mapping to AA

The reacted thermoset structures were reverse-mapped from CG Martini to the atomistic level for more detailed thermo-mechanical analyses. First, the topology file of the CG structures was converted to an atomistic OPLS-AA forcefield through a developed scripting procedure. Then, the structure files of atomistic level were produced utilizing the backward mapping approach of Martini.[8] To achieve a proper structure with the right positions of the atoms, a multi-step relaxation procedure was performed on the output structures. An energy minimization step followed by four relaxation simulations (500,000 steps with 0.1, 0.2, 0.5, and 1 fs, respectively) were performed in the NVT ensemble using a V-rescale thermostat and constraints on all bonds. The output structure and topology files were used for the simulation of thermo-mechanical properties.

To ensure the accuracy and validation of the reverse mapping process, we used a graph-based analysis[9] that classifies all the clusters in the system based on the connectivity of each atom or bead. Note that the spatial arrangement of the beads is not retained in the constructed graphs, and they are represented only based on the connectivity information taken from the structural information. The analysis was performed on a representative sample (L-F3 system at conversion 24%) and the results are shown in the Figure S10. As shown, the clusters before and after reverse mapping are identical. In addition, the number of clusters in the CG and atomistic systems was also extracted and compared separately. This comparison showed that the number of clusters in the reverse-mapped systems is completely equal to that in the CG ones. This finding is critical to reconfirm the correctness of the connectivity information, including both intra-cluster and inter-cluster linkages, upon reverse-mapping.

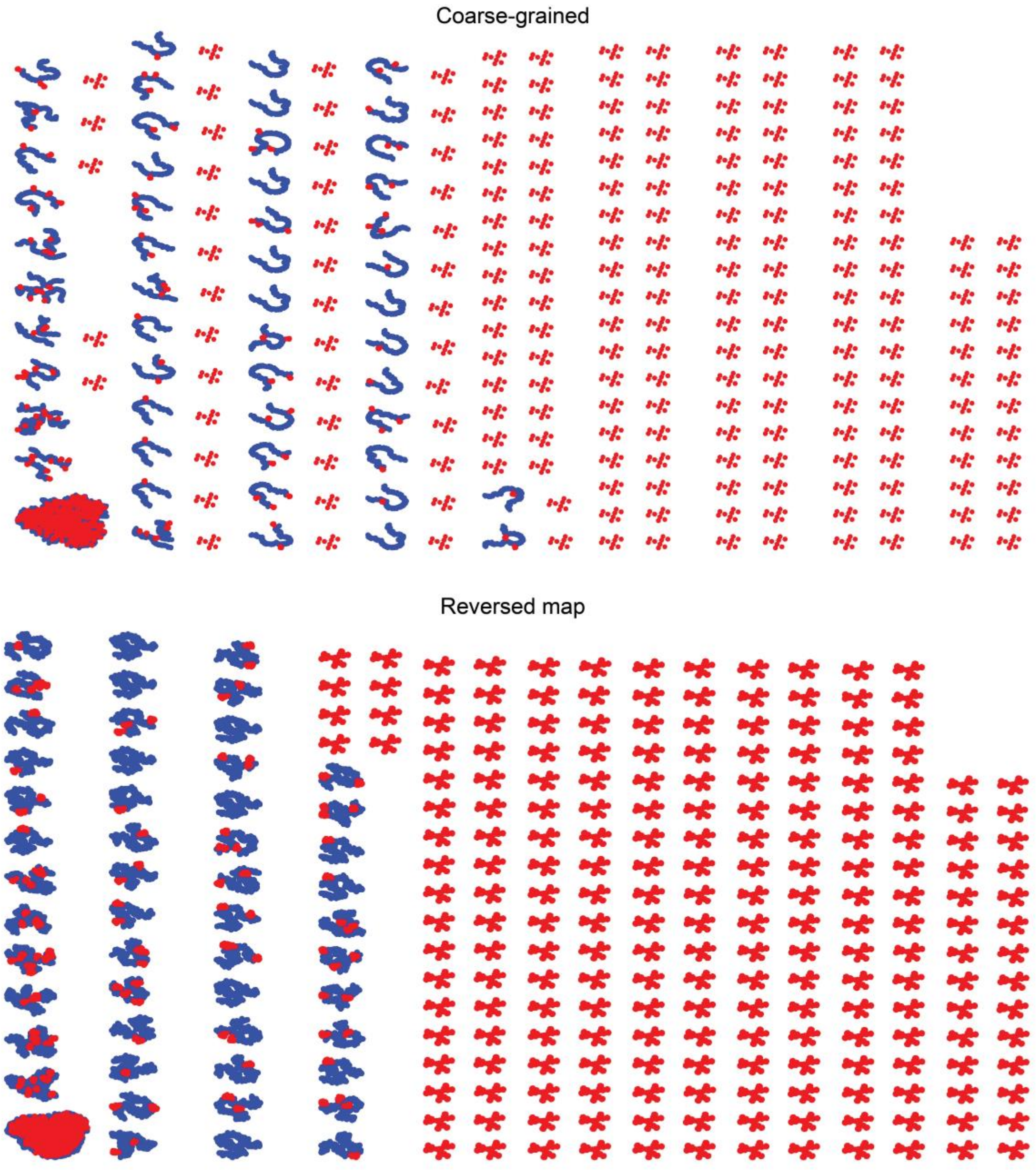

Figure S10. Cluster analysis of the polymer system at two scales: coarse-grained (top) and reverse-mapped atomic scale (bottom).

To achieve complete relaxation and ensure system stability, the relaxation process was performed gradually over two iterations. This relaxation involved cooling the system from 800 K to 300 K over a period of 100 ns. The density of equilibrated network at CG and atomistic levels are 1.182 and 1.108 g/cm³m respectively. In addition, Figure S11 shows the distribution of cross-linkers in the CG and equilibrated atomistic model. To gain a quantitative picture, the distribution of cross-linkers can be analyzed using the second virial coefficient (B_2) and the radial distribution function (RDF) of the cross-linkers. B_2 is an index for the uniformity of the distribution of cross-linkers and is defined by the equation S6:[10]

$$B_2 = \frac{1}{2}\int_0^\infty [\exp(g(r)\text{-}1))]\ 4\pi r^2 dr \qquad \text{(SE6)}$$

where g(r) is the radial distribution function. A positive B_2 value indicates a uniform distribution, and a negative B_2 value indicates clustering or aggregation of cross-linkers. The B_2 values for cross-linkers in both atomistic and CG levels are positive and approximately equal to 1252, indicating a uniform distribution. However, RDF for the melamines in both scales shows some degree of local

aggregations (see Figure 1d in the paper).

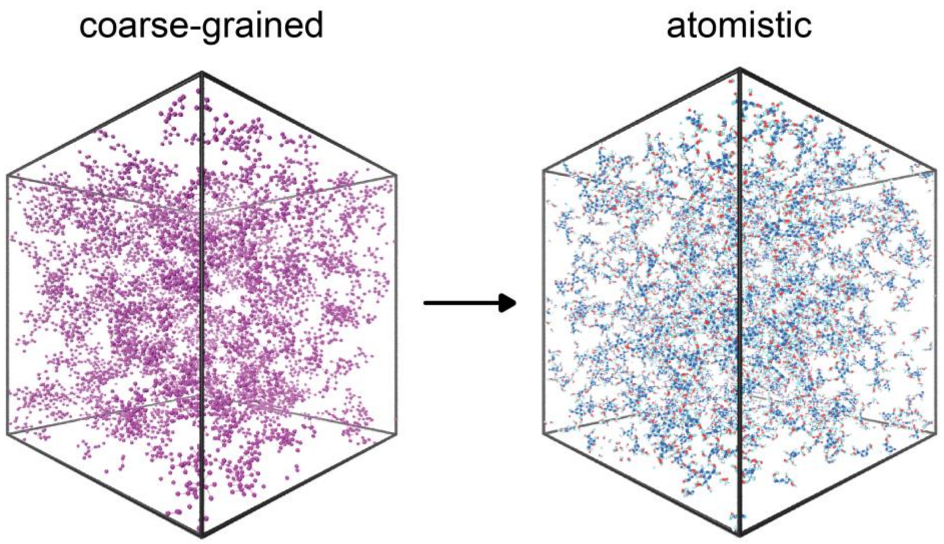

Figure S11. Comparison of cross-linker distribution in coarse-grained (left) and atomistic (right) representations

S.2.7. Calculation of mechanical properties and T_g in simulation

S2.7.1 Tensile simulation

To simulate the tensile test in MD, the reverse-mapped systems were stretched in the Z direction by keeping the pressure constant (1 bar) in the X and Y directions at the temperature of 300 K. V-rescale thermostat and Parinello-Rahman barostat was used with a time step of 2 fs and a strain rate of 10^8 s^{-1}. Elastic modulus was obtained from fitting data in 2-5 % of strain in the linear portion of the stress-strain curve (Figure S12). Due to stress fluctuations at the onset of stretching, initial stress values may include some error, causing the graph not to pass through the origin (0,0). To address this, the analysis excluded the initial region and focused on the range starting from 2% strain to 5%, where the curve exhibits approximately linear behavior. It should be noted that the force field limitation does not allow bond breakage in MD. Therefore, we have limited the analysis only to the linear elastic region. To ensure the accuracy of the results, 5 repetitions were performed for each sample and the average results were reported along with the standard deviation. On average, atomistic systems have nearly 300,000 atoms, which is an acceptable amount according to Li and Strachan's study[11] (systems with sizes from 16000 to 65000 atoms showed little dependence on size for mechanical behavior). This large system size reduces the stress fluctuations during stretching and improves the accuracy of the results obtained from the tensile test.

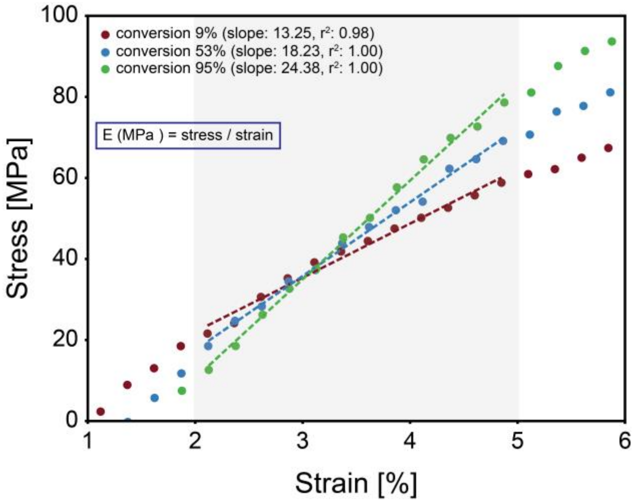

Figure S12. An example of stress-strain curves at different conversion levels. Linear elastic region (shaded) used for calculating the elastic modulus.

S2.7.2 T_g calculation

The glass transition temperature is a kinetic phenomenon where the molecular mobility of the system begins to change dramatically. As temperature decreases, molecular motion slows down until it reaches a point where large-scale molecular rearrangements become very slow or effectively stop on observable time scales. Therefore, T_g can be estimated by the calculation of mean square displacement (MSD) of atoms, which is the measurement of the average distance that the particles of a system travel over time, during successive annealing cycles, from a temperature much higher than the T_g to a much lower temperature. In this work, we initiated cooling from 620 K to 240 K with 20 K intervals and 2 ns relaxation in each temperature. Since the T_g is sensitive to the cooling rate, the same conditions were applied to all systems. Above the T_g, molecular segments have high mobility, which leads to an increase in MSD value. Conversely, below the T_g, molecular motion is largely limited to oscillations around fixed positions, which leads to a plateau of MSD at very low values. Consequently, MSD can be utilized to directly investigate the dynamics and molecular movement of chains, which is closely related to the definition of T_g.

In this work, the MSD value (in nm^2/ns) was calculated at the end of each cooling cycle (values at 2 ns for each cycle) and plotted against temperature. A cubic spline was then fitted to data to provide a smooth approximation of the relationship between temperature and MSD (Figure S13).

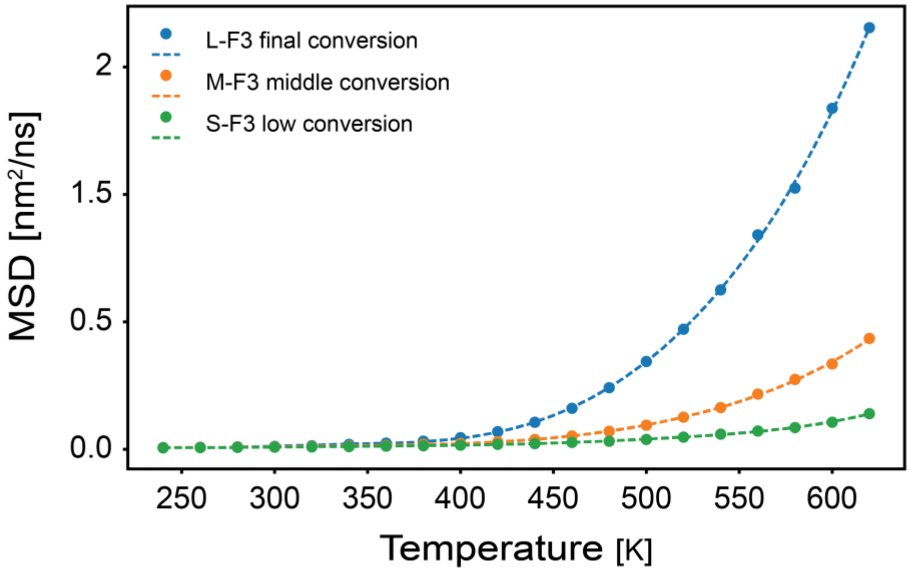

Figure S13. Temperature dependence of MSD for systems at different conversion levels: L-F3 (high conversion, blue), M-F3 (middle conversion, orange), and S-F3 (low conversion, green). Dashed lines show fitted cubic splines.

After that, interpolation is used to find a temperature corresponding to a specific target MSD value. The MSD of the L-F3 system with 85% conversion at the determined T_g in DSC analysis was considered as the reference. For other systems, when the MSD reaches this same reference value, the corresponding temperature is identified as the glass transition temperature (Figure S14). This method (i) avoids inaccuracies associated with very high temperature simulations needed for T_g calculations from volume-temperature curves, (ii) uncertainties related to the fitting procedure, and (iii) avoids ambiguous conversion relations between simulation and experimental T_g calculations.[12,13]

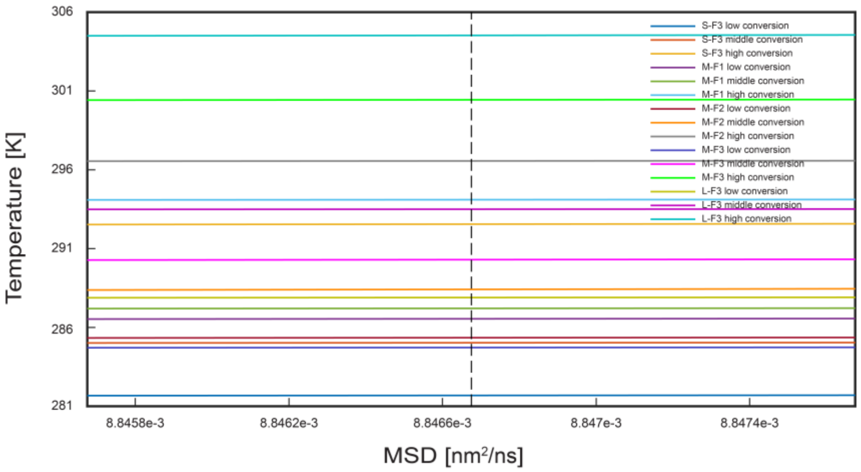

Figure S14. Temperature versus MSD for all systems at different conversion (low, middle, and high). The dashed vertical line indicates the reference MSD point for comparison across systems. The intersection of the dashed line with each colored line determines the T_g.

In addition to the MSD method, the results from conventional approach such as volume-temperature (V-T) curves were examined (Figure S15). Initially, three points from the beginning and three points from the end of the V-T curve are selected for fitting, and two linear lines are fitted on these points and the intersection of these lines is determined as T_g. To explore the effect of the fitting range, the number of data points used is progressively increased (up to nine initial and terminal points). The results show that in the V-T method, T_g value strongly depends on the fitting method (Figure S16). An alternative approach utilized a hyperbolic fitting model, as suggested by Ref [12], in which all data points from the V-T curve were incorporated into the fitting process. This method shows better results than the linear method and is able to predict the overall trend of the results with higher accuracy. However, the problem with these two methods, apart from their lack of accuracy, is that the results obtained do not quantitatively agree with the experimental data because of the non-linear relationship between the estimated T_g and heating rate and significantly high heating rates used in simulations.

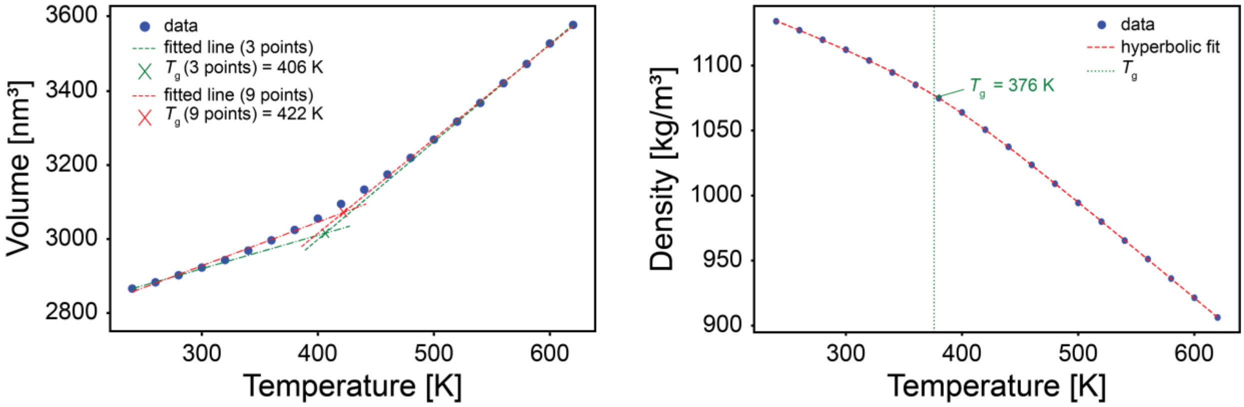

Figure S15. Comparison of T_g determination methods: (left) Volume vs. temperature with linear fitting applied to three and nine data points. (right) Density vs. temperature with a hyperbolic fit.

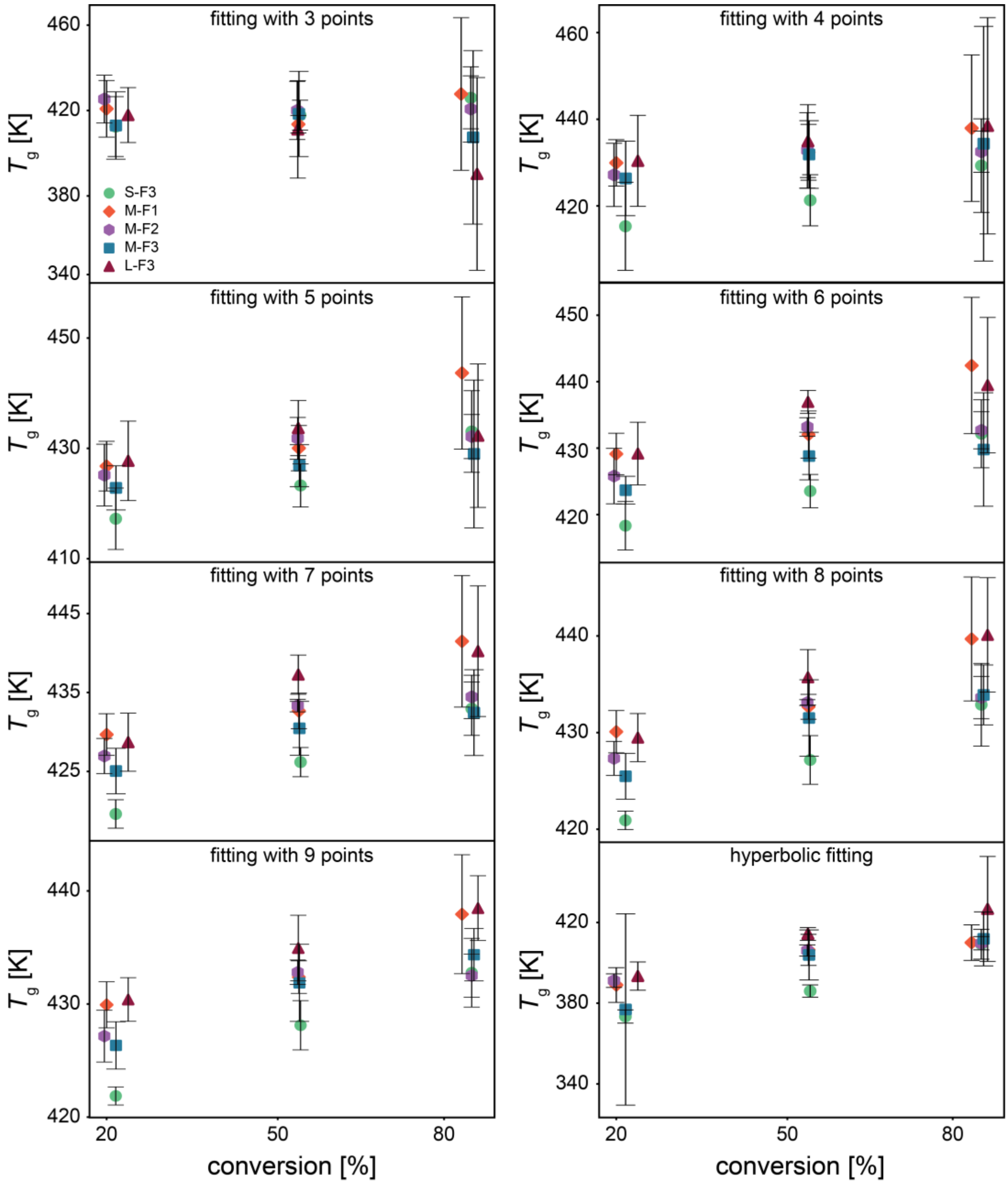

Figure S16. Effect of data point selection and fitting methods on the determination of T_g at different conversion levels. T_g values are shown for linear fitting using 3 to 9 points (top to bottom) and hyperbolic fitting (bottom-right).

Our proposed method is based on the direct analysis of molecular mobilities through MSD. In this method, instead of relying on secondary properties such as volume or density, the dynamic behavior of molecules is investigated directly. This method, has the ability to predict the trend of the results and the resulting values also agree well with the experimental data obtained from DSC. To further validate our method, the MSD of specific molecular components—such as prepolymers and the repeating blocks of monomers—was also calculated (Figure S17). These subsystem MSD values exhibited strong correlations with the MSD of the entire system. While slight differences in the exact T_g values are observed, the overall consistency and reliability of the method are reaffirmed.

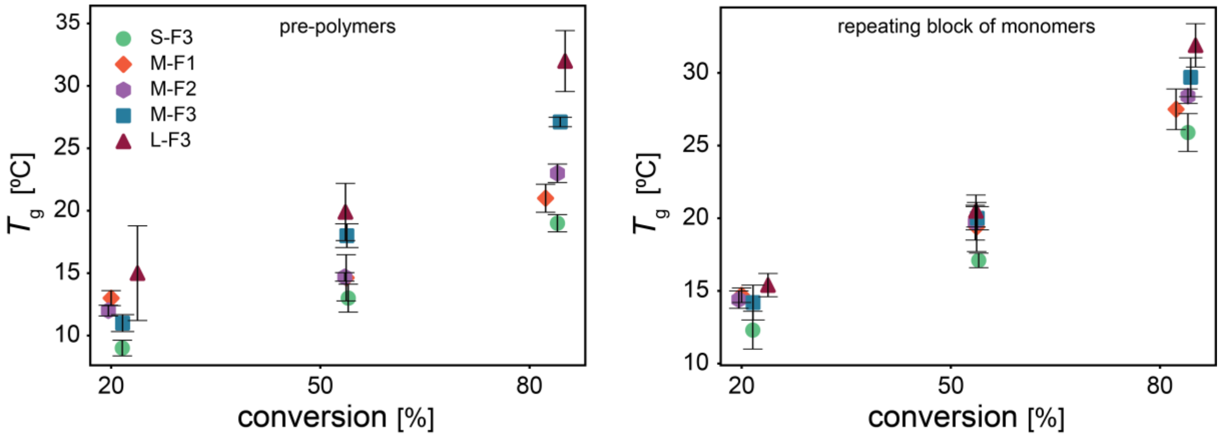

Figure S17. T_g as a function of conversion based on the MSD of the center of mass of prepolymers (left) and repeating blocks of monomers (right).

All in all, the results of T_g calculated by different methods for L-F3 system confirm the increasing trend of T_g with increasing conversion, which is also observed in experimental samples (Figure S4). However, the MSD method shows a better quantitative agreement with the experimental data and provides more accurate results. Although the MSD method is calibrated with an experimental data, the variation of the other calculated points remains within the experimental range and maintains the correct trend.

S3. Results

S3.1. Conversion growth with reaction progress

Conversion of each reactive group is determined by dividing the number of reacted beads by the total number of available beads for that group. The weighted average conversion with respect to both hydroxyl and carboxyl groups is defined as the reference conversion in this work. As expected from step-growth polymerization reactions, an initial rapid growth in conversion is exhibited by all systems. As the reaction progresses, the mobility of the system decreases because of the increasing connections between polymer chains, which make the reactive groups less available and the reaction speed decreases. Since the ratio of hydroxyl to carboxyl groups is the same, the conversion in S-F3, M-F3, and L-F3 systems are similar (Figure S18). However, when the number of hydroxyl groups changes in the M-F2 and S-F1 systems, differences become apparent in the intermediate conversions due to the varying total functionality of the polymer chains.

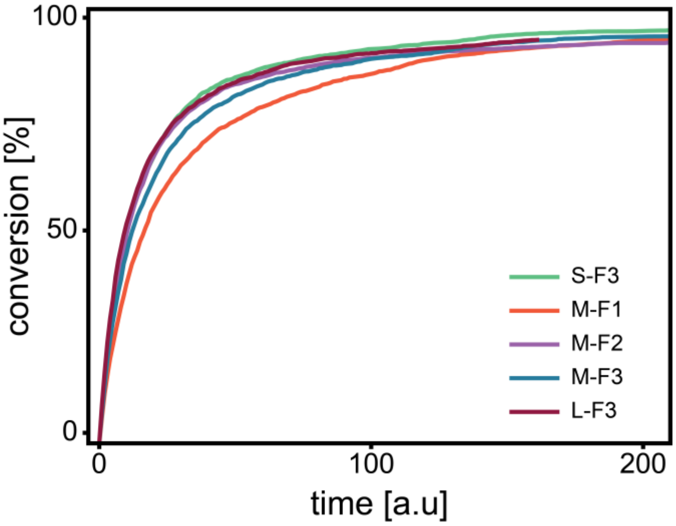

Figure S18. Conversion growth over time.

S3.2. Evolution of different types of partially-reacted melamines

The number of junctions for melamines during the reaction follows a similar trend across all systems (Figure S19). The number of unreacted melamines decreases continuously, starting with a high rate of reduction that gradually slows until all melamines participate in reactions. In contrast, the number of melamines with five or six junctions shows an opposite trend. They do not appear until intermediate conversions and then their number increases rapidly. Melamines with one, two, and three junctions exhibit different behaviors. Peak for melamine with one junction occurs at lower conversions, while melamines with two junction peaks at medium conversions, and three-bonded melamines peak at higher conversions. This sequential peaking of different partially-reacted species shows that as the reaction progresses, these molecules participate more in the reaction, which leads to a decrease in the number of melamines with fewer bonds and an increase in melamines with higher bonds. By the final stages of the reaction, most of the melamines have at least four junctions.

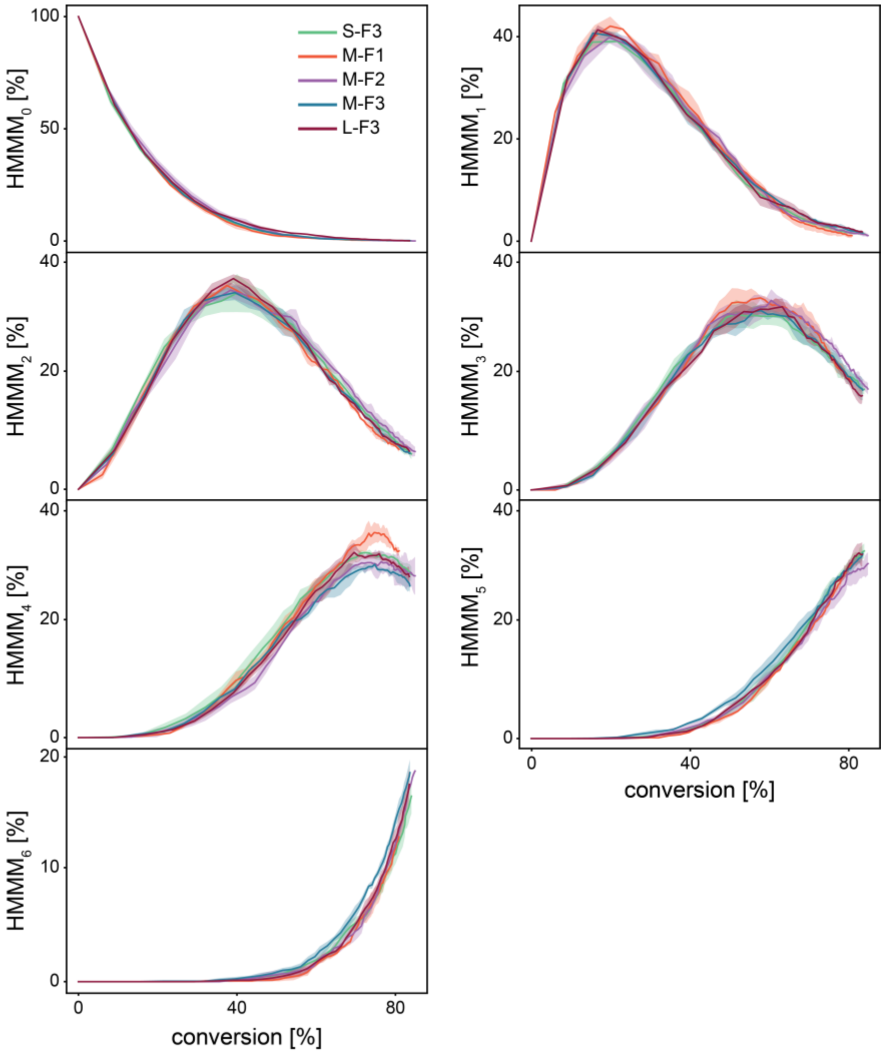

Figure S19. Trend of different types of melamines based on the number of junctions during the reaction. The subscript shows the number of junctions for melamines

S3.3. Evolution of clusters during the reaction

Having the connectivity information of beads, we conducted cluster analysis using graph theory and the network algorithm library to visualize the molecular structure of the systems. Using this tool, each cluster in the system is identified and all clusters are sorted based on the number of beads they contain (Figure S20). The important thing to note in this methodology is that the spatial arrangement of the beads is not retained, and they are represented only based on their connectivity.

As shown in Figure S20, for L-F3 system, even at initial conversions, a large portion of the chains are connected to each other. For example, at 34% conversion, only seven chains remain unconnected to the network. In contrast, at this same conversion in S-F3 system, the network has not yet been formed, and the largest cluster consists of very few acrylic chains. Even at 85% conversion in S-F3 system, some of the chains have not reacted even once and remain free from the network. However, in L-F3 system, all polymers and cross-linkers have participated in the network. These results visually illustrate the growth of the clusters, indicating that with an increase in the chain length and functionality of the prepolymer, due to the presence of more potential connection sites, the network begins to form at lower conversions.

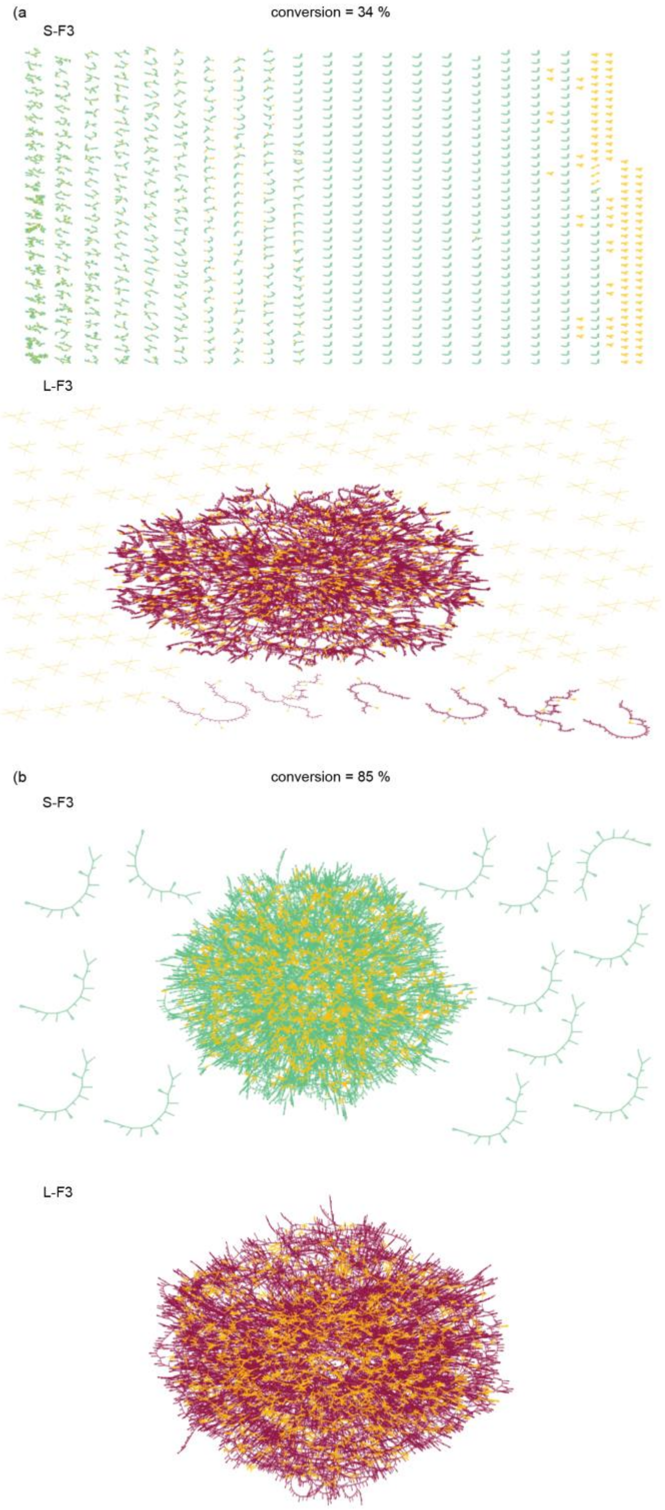

Figure S20. Comparison of clusters formed at 34% and 85% conversions for S-F3 and L-F3 systems. Melamines are shown in orange, S-F3 system in green and L-F3 systems in red. Note that the spatial position of the molecules is not

maintained and the display criterion is only based on the connection of the molecules to each other.

S3.4. Gelation point determination

The gelation point was determined using different approaches (Figure S21). The first method is to examine the molecular weight of the whole system. The inflection point of molecular weight versus conversion is where a sudden change in the rate of increase in molecular weight occurs, called the gelation point. This point marks the transition from a viscous liquid to a gel-like network. With the increase of the molecular weight of acrylic prepolymer, due to the earlier formation of the network, the gelation point occurs at a lower conversion (42%, 30%, and 20% conversions for S-F3, M-F3, and L-F3 systems, respectively). Increasing the functionality of prepolymer also has the same effect as increasing the chain length and shifts the gelation point to lower conversions. However, the effect of chain length is significantly more prominent. Another way to determine the gelation point is reduced molecular weight (RMW) which is defined as the average molecular weight of all formed clusters except the largest cluster. The evolution of RMW is similar to the second largest cluster and the maximum point shows the gelation.

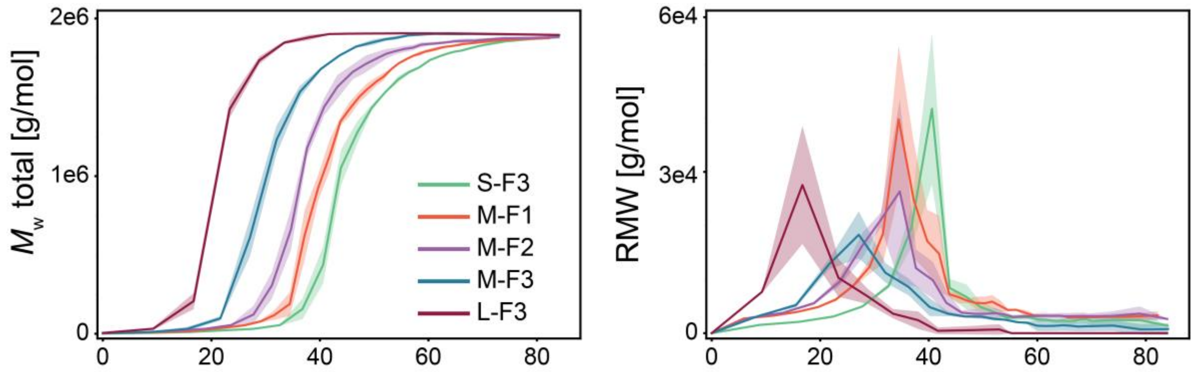

Figure S21. Evolution of the M_W of the whole system and RMW as a function of conversion for different systems.

S3.5. Formation of loops with different complexity

Considering that melamine has six functional groups, the resulting loops can also have some degree of complexity. Loops can be formed by connecting two to six functionalities of a single melamine to a single acrylic chain. As a result, they can be categorized into five different types. We named them $loop_2$, $loop_3$, $loop_4$, $loop_5$, and $loop_6$ (for example, $loop_6$ indicates that all six functionalities of a melamine reacted with a single acrylic chain) and Figure S22. depicts the trend of loop formation during the reaction. More complex loops (such as $loop_5$ and $loop_6$) appear in systems with higher chain lengths, only at final conversions. However, even at 85% conversion, there is a very small amount of $loop_6$ (0.04%) solely for the L-F3 system. Simpler loops, like $loop_2$, begin to form early in the reaction, while $loop_3$ gradually emerges as the reaction progresses. Similarly, $loop_4$ and $loop_5$ appear progressively as the reaction continues. The formation of loops with more complexity reduces the possibility of a melamine to connect different acrylic chains resulted in a reduced possibility of encountering more elastically effective melamines as a cross-link point. Consequently, as shown in Figure S23, the percentage of more elastically effective melamines (as an example, melamines that connect to 5 or 6 different acrylics calculated) increases with decreasing chain length and functionality.

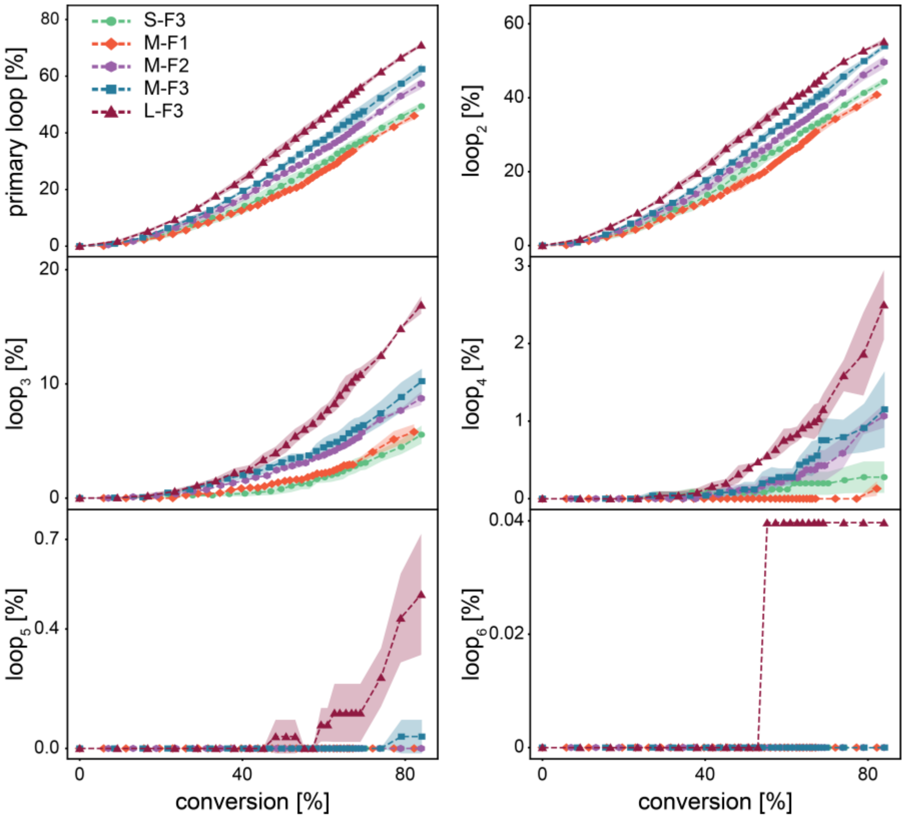

Figure S22. Percentage of melamines that formed primary loop and loops with different complexity during the reaction for each system. The subscripts show the number of junctions that a melamine formed with a single acrylic chain.

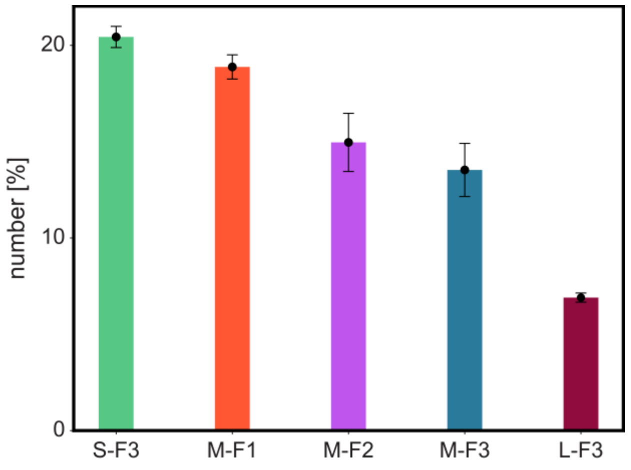

Figure S23. Percentage of melamines with 5 or 6 connections to different acrylics at conversion 85%.

S3.6. Stress-strain curves at larger strains

The modeled samples in MD were stretched to 100% strain to simulate the tensile behavior of the networks. Since the bonds are not broken in our force field, no fracture occurs in the samples. As a result, analyzing high strains in the simulation does not accurately reflect physical reality. However, plastic deformation is observed in the form of chain sliding and rearrangement, which makes it possible to estimate the relative strength.

There is a notable difference in behavior at high strains across the various systems Figure S24. As the conversion increases, the stress-strain curves shift upwards for all modeled networks. Consequently, the stress at final strains increases with the increase of conversion. This trend indicates a higher conversion leads to stronger networks with greater resistance to deformation, which is consistent with the results of the DMA analysis. After the initial elastic response, the systems display slight softening behavior at lower conversion and strain hardening at higher

conversion. At lower degrees of cross-linking, the molecular chains are more mobile, which facilitates the rearrangement in response to the applied stress and absorbs displacement energy. Conversely, systems with high cross-linking are less flexible having limited rearrangement and sliding to absorb stress. This causes a continuous increase in stress with increasing strain on these systems. At low conversion (up to 34%), the stress-strain curves are relatively flat, which indicates a more flexible network due to less cross-linking density. As the conversion increases (51-64%), a sharper curve is observed that shows the onset of strain hardening. At higher conversions (85–95%), the curves become significantly steeper, especially at higher strains. This shows the formation of a network with a very high level of connectivity.

Another aspect to consider is the stress difference at ultimate strains for the different systems. At equal conversion, a higher chain length of acrylic prepolymer, has a higher amount of stress at the final strains. For instance, at 95% conversion, the stress at the ultimate strain for S-F3, M-F3 and L-F3 systems is 2000, 3700, and 4500 MPa, respectively. This increase in stress attributes to the formation of a stronger network with more connections. Therefore, examining the stress of samples at high strains can be used as a complementary method to evaluate the strength of networks.

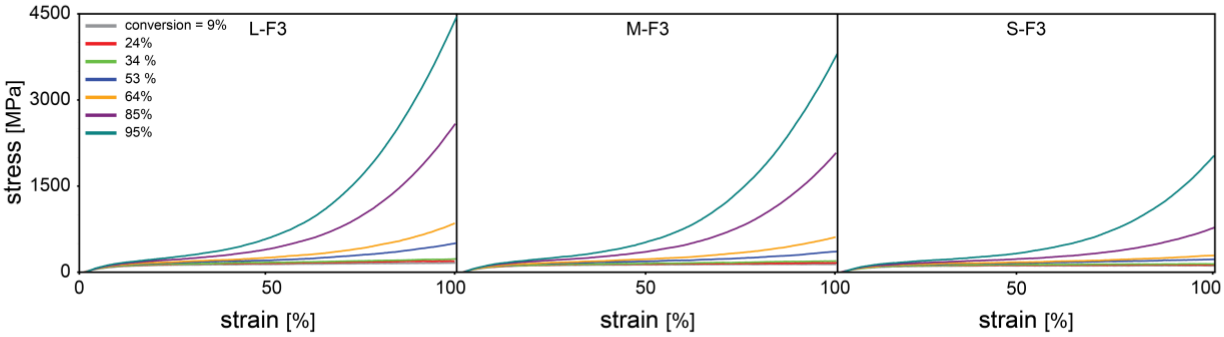

Figure S24. Strain-stress curves at different conversions for systems with different chain lengths until 100% strain.

S3.7. Representative Calculation of XLD^{eff} for the L-F3 Model at 70% Conversion

To clarify the implementation of our modified cross-link density (XLD^{eff}), we provide a representative case study for the L-F3 model at 70% conversion. This example illustrates the three-step approach described in Section 3.2 of the main manuscript, which refines the conventional XLD by incorporating the elastic effectiveness of each junction point in the polymer network.

In the first step, we identify elastically effective junctions along the prepolymer chains—those that connect to the network through three distinct paths. These points typically occur along branched segments and exclude chain ends or functionalities that result in dangling chains. In this snapshot, 980 such junctions were found, each connecting two different molecules (a cross-linker and a prepolymer), and thus assigned an elasticity coefficient of 2.

In the second step, we consider the unequal elastic contributions of melamine cross-linkers by categorizing them based on the number of distinct chains to which they are connected. In this case, 306 melamine molecules connect to three distinct chains, 150 to four, 24 to five, and 1 to six chains. Each of these is assigned a coefficient equal to its connectivity (3 to 6, respectively).

The third correction accounts for the elastic contribution of *longer* the prepolymers. Each long chain contains three hypothetical link that connect four reference chains. With 300 long chains, this adds 900 hypothetical links. Since each hypothetical link connects two distinct molecular units, we assign them a coefficient of 2. A summary of these values is provided in Table S11.

The total weighted number of elastically effective cross-link points, N_{eff}, can be calculated as:

$$N_{eff} = N_{hyp} \times 2 + N_{branch} \times 2 + N_3 \times 3 + N_4 \times 4 + N_5 \times 5 + N_6 \times 6 \quad \text{(SE7)}$$

Where:

- N_{hyp} is the total number of hypothetical links within the system,
- N_{cl} is the total number of cross-link points on prepolymer chains,
- N_3, N_4, N_5, N_6, are the numbers of melamine cross-linkers connected to 3, 4, 5, and 6 distinct chains, respectively.

Once N_{eff} is determined, the XLD^{eff} is calculated as:

$$XLD^{eff} = \frac{N_{eff}}{6.023\times10^{23}\,(\text{mol}^{-1}) \times V\,(\text{m}^3)} \qquad \text{(SE8)}$$

Where V is the simulation box volume.

Consequently, for the L-F3 system at 70% conversion, XLD^{eff} is calculated as:

$$N_{eff} = 900\times2 + 980\times2 + 306\times3 + 150\times4 + 24\times5 + 1\times6 = 5404$$

$$XLD^{eff} = \frac{5404}{6.022\times10^{23}\,(\text{mol}^{-1}) \times 2786.6\times10^{-27}\,(\text{m}^3)} = 3.22\times10^{3}\,\left(\frac{\text{mol}}{\text{m}^3}\right)$$

Table S11. Summary of cross-link types and their counts for the L-F3 model at 70% conversion.

Cross-link type	N_{hyp}	N_{cl}	N_3	N_4	N_5	N_6
Number	900	980	306	150	24	1

References

(1) Jorgensen, W. L.; Tirado-Rives, J. The OPLS Potential Functions for Proteins. Energy Minimizations for Crystals of Cyclic Peptides and Crambin. *J. Am. Chem. Soc.* **1988**, *110* (6), 1657–1666. https://doi.org/10.1021/ja00214a001.

(2) Bussi, G.; Donadio, D.; Parrinello, M. Canonical Sampling through Velocity Rescaling. *J. Chem. Phys.* **2007**, *126* (1). https://doi.org/10.1063/1.2408420.

(3) Bernetti, M.; Bussi, G. Pressure Control Using Stochastic Cell Rescaling. *J. Chem. Phys.* **2020**, *153* (11). https://doi.org/10.1063/5.0020514.

(4) Souza, P. C. T.; Alessandri, R.; Barnoud, J.; Thallmair, S.; Faustino, I.; Grünewald, F.; Patmanidis, I.; Abdizadeh, H.; Bruininks, B. M. H.; Wassenaar, T. A.; Kroon, P. C.; Melcr, J.; Nieto, V.; Corradi, V.; Khan, H. M.; Domański, J.; Javanainen, M.; Martinez-Seara, H.; Reuter, N.; Best, R. B.; Vattulainen, I.; Monticelli, L.; Periole, X.; Tieleman, D. P.; de Vries, A. H.; Marrink, S. J. Martini 3: A General Purpose Force Field for Coarse-Grained Molecular Dynamics. *Nat. Methods* **2021**, *18* (4), 382–388. https://doi.org/10.1038/s41592-021-01098-3.

(5) Konishi, T.; Yoshizaki, T.; Saito, T.; Einaga, Y.; Yamakawa, H. Mean-Square Radius of Gyration of Oligo- and Polystyrenes in Dilute Solutions. *Macromolecules* **1990**, *23* (1), 290–297. https://doi.org/10.1021/ma00203a050.

(6) Mousavifard, S. M.; Ghermezcheshme, H.; Mirzaalipour, A.; Mohseni, M.; de With, G.; Makki, H. PolySMart: A General Coarse-Grained Molecular Dynamics Polymerization Scheme. *Mater. Horizons* **2023**, *10* (6), 2281–2296. https://doi.org/10.1039/d3mh00088e.

(7) Bauer, D. R.; Dickie, R. A. Crosslinking Chemistry and Network Structure in Organic Coatings - 2. Effect of Catalysts on Cure of Melamine Formaldehyde/Acrylic Copolymer Films. *J. Polym. Sci. Part A-2, Polym. Phys.* **1980**, *18* (10), 2015–2025. https://doi.org/10.1002/pol.1980.180181002.

(8) Wassenaar, T. A.; Pluhackova, K.; Böckmann, R. A.; Marrink, S. J.; Tieleman, D. P. Going Backward: A Flexible Geometric Approach to Reverse Transformation from Coarse Grained to Atomistic Models. *J. Chem. Theory Comput.* **2014**, *10* (2), 676–690. https://doi.org/10.1021/ct400617g.

(9) Ghermezcheshme, H.; Makki, H.; Mohseni, M.; Ebrahimi, M.; De With, G. MARTINI-Based Simulation Method for Step-Growth Polymerization and Its Analysis by Size Exclusion Characterization: A Case Study of Cross-Linked Polyurethane. *Phys. Chem. Chem. Phys.* **2019**, *21* (38), 21603–21614. https://doi.org/10.1039/c9cp03407b.

(10) Liu, J.; Gao, Y.; Cao, D.; Zhang, L.; Guo, Z. Nanoparticle Dispersion and Aggregation in Polymer Nanocomposites: Insights from Molecular Dynamics Simulation. *Langmuir* **2011**, *27* (12), 7926–7933. https://doi.org/10.1021/la201073m.

(11) Li, C.; Strachan, A. Evolution of Network Topology of Bifunctional Epoxy Thermosets during Cure and Its Relationship to Thermo-Mechanical Properties: A Molecular Dynamics Study. *Polymer (Guildf).* **2015**, *75*, 151–160. https://doi.org/10.1016/j.polymer.2015.08.037.

(12) Patrone, P. N.; Dienstfrey, A.; Browning, A. R.; Tucker, S.; Christensen, S. Uncertainty Quantification in Molecular Dynamics Studies of the Glass Transition Temperature. *Polymer (Guildf).* **2016**, *87*, 246–259. https://doi.org/10.1016/j.polymer.2016.01.074.

(13) Afzal, M. A. F.; Browning, A. R.; Goldberg, A.; Halls, M. D.; Gavartin, J. L.; Morisato, T.; Hughes, T. F.; Giesen, D. J.; Goose, J. E. High-Throughput Molecular Dynamics Simulations and Validation of Thermophysical Properties of Polymers for Various Applications. *ACS Appl. Polym. Mater.* **2021**, *3* (2), 620–630. https://doi.org/10.1021/acsapm.0c00524.
